# Brain Tumor Diagnosis Using Machine Learning, Convolutional Neural Networks, Capsule Neural Networks and Vision Transformers, Applied to MRI: A Survey

**DOI:** 10.3390/jimaging8080205

**Published:** 2022-07-22

**Authors:** Andronicus A. Akinyelu, Fulvio Zaccagna, James T. Grist, Mauro Castelli, Leonardo Rundo

**Affiliations:** 1 NOVA Information Management School (NOVA IMS), Universidade NOVA de Lisboa, Campus de Campolide, 1070-312 Lisboa, Portugal; mcastelli@novaims.unl.pt; 2 Department of Computer Science and Informatics, University of the Free State, Phuthaditjhaba 9866, South Africa; 3 Department of Biomedical and Neuromotor Sciences, Alma Mater Studiorum-University of Bologna, 40138 Bologna, Italy; fulvio.zaccagna@unibo.it; 4 IRCCS Istituto delle Scienze Neurologiche di Bologna, Functional and Molecular Neuroimaging Unit, 40139 Bologna, Italy; 5 Department of Physiology, Anatomy, and Genetics, University of Oxford, Oxford OX1 3PT, UK; james.grist@dpag.ox.ac.uk; 6 Department of Radiology, Oxford University Hospitals NHS Foundation Trust, Oxford OX3 9DU, UK; 7 Oxford Centre for Clinical Magnetic Research Imaging, University of Oxford, Oxford OX3 9DU, UK; 8 Institute of Cancer and Genomic Sciences, University of Birmingham, Birmingham B15 2SY, UK; 9 Department of Information and Electrical Engineering and Applied Mathematics, University of Salerno, 84084 Fisciano, Italy

**Keywords:** brain cancer, magnetic resonance imaging, machine learning, deep learning, capsule neural networks, vision transformers

## Abstract

Management of brain tumors is based on clinical and radiological information with presumed grade dictating treatment. Hence, a non-invasive assessment of tumor grade is of paramount importance to choose the best treatment plan. Convolutional Neural Networks (CNNs) represent one of the effective Deep Learning (DL)-based techniques that have been used for brain tumor diagnosis. However, they are unable to handle input modifications effectively. Capsule neural networks (CapsNets) are a novel type of machine learning (ML) architecture that was recently developed to address the drawbacks of CNNs. CapsNets are resistant to rotations and affine translations, which is beneficial when processing medical imaging datasets. Moreover, Vision Transformers (ViT)-based solutions have been very recently proposed to address the issue of long-range dependency in CNNs. This survey provides a comprehensive overview of brain tumor classification and segmentation techniques, with a focus on ML-based, CNN-based, CapsNet-based, and ViT-based techniques. The survey highlights the fundamental contributions of recent studies and the performance of state-of-the-art techniques. Moreover, we present an in-depth discussion of crucial issues and open challenges. We also identify some key limitations and promising future research directions. We envisage that this survey shall serve as a good springboard for further study.

## 1. Introduction

Brain tumors are expansile lesions that originate in the brain [1,2]. They can be divided into benign (non-cancerous) and malignant (cancerous) according to their aggressiveness, and classified into four grades using the WHO classification for Central Nervous System (CNS) tumors ranging from 1 to 4, according to their malignancy [1]. Non-cancerous tumors rarely spread to healthy surrounding cells, examples are meningiomas and pituitary tumors, often of lower grades [3]. Malignant brain tumors infiltrate the surrounding parenchyma with variable aggressiveness. Glioblastoma (GBM) is the most common and aggressive type of malignant brain tumor, commonly classified as a grade 4 CNS tumor with dismal survival [3,4]. Brain tumors can also be categorized according to their origin into primary and secondary brain tumors, with the former originating in the brain and the latter usually in a distant site [3].

At present, diagnosis and management are based upon clinical and radiological information. Magnetic Resonance Imaging (MRI) is the mainstay for the assessment of patients with brain tumors [5], although conventional imaging has significant limitations in evaluating tumor extent, predicting grade, and assessing treatment response [6]. Novel acquisition techniques are under development to improve lesion characterization, therapy assessment and management [7]; however, novel approaches to image analysis have been gaining traction thanks to the wealth of information possessed by radiological images [8].

In this context, brain tumor classification and segmentation have become pivotal for image analysis. Brain tumor classification can be performed with different methods, including manual classification and computer-aided classification. Manual classification of brain tumors is very time-consuming [9], and prone to error [10]. However, manual classification cannot be ignored, as it is still the reference standard both for clinical care and used as a comparison for other techniques [11].

Computer-aided diagnosis has proven to be useful in supporting medical practitioners [12]. It can be performed using different techniques including classical machine learning (ML) [13,14] (such as clustering [15]), Convolutional Neural Network (CNN) approaches [16,17], recently considering also capsule neural networks (CapsNets) [18,19], and Vision Transformers (ViTs) [20].

This paper presents a survey on brain tumor segmentation and classification techniques, with a particular emphasis on ML-based, CNN-based, CapsNet-based, and ViT-based techniques.

The primary objective of this survey is to highlight the current state-of-the-art of ML-based, CNN-based, CapsNet-based, and ViT-based brain tumor segmentation and classification methods. This paper also highlights recent achievements, relevant research challenges, and future research directions. This survey is different from the existing surveys [9,21,22,23] in the following ways:

1. Most review papers considered one or two types of ML-based techniques, while we included four types of brain tumor segmentation and classification techniques: classical ML algorithms, CNN-based techniques, CapsNet-based techniques, and ViT-based techniques. This survey summarizes the current state-of-the-art in brain tumor segmentation, grade estimation, and classification. It outlines the advances made in ML-based, CNN-based, CapsNet-based, and ViT-based brain tumor diagnosis between 2019 and 2022.
2. Most of the previous studies [9,21,22,23] presented an overview of either CNN-based techniques, ML-based techniques, or both ML- and CNN-based approaches. In addition to CNN-based and ML-based techniques, we present a summary of CapsNet-based, and ViT-based brain tumor segmentation and classification techniques. CapsNet is one of the state-of-the-art techniques for brain tumor segmentation and classification. The findings of this survey show that CapsNet outperformed CNN-based brain tumor diagnosis techniques, as they require significantly less training data compared to CNNs. Moreover, CapsNets are very robust to different rotations and image transformations. Furthermore, unlike CNN, ViT-based models can effectively model local and global feature information, which is very critical for accurate brain tumor segmentation and classification.
3. Some review papers did not provide a comprehensive discussion and pertinent findings. This survey presents significant findings and a comprehensive discussion of approaches for segmenting and classifying brain tumors. We also identify open problems and significant areas for future research, which should be beneficial to interested scholars and medical practitioners.

The remainder of this manuscript is structured as follows: Section 2 introduces some fundamental concepts necessary for an accurate understanding of this manuscript. Section 3 introduces the material and methods used for this survey. Section 4 provides an in-depth review of recently published ML, CNN, CapsNet, and ViT-based brain tumor classification and segmentation techniques. Section 4 also highlights the core contributions and performance of various studies. Section 5 discusses several open research problems and critical issues related to brain tumor segmentation and classification. Section 5 also presents a performance analysis of brain tumor diagnosis techniques, as well as details on several popular datasets. Section 6 concludes the paper with some final thoughts and potential research directions.

## 2. Background

### 2.1. Brain Tumors and Magnetic Resonance Imaging

Brain tumors can be intra-axial (e.g., gliomas) or extra-axial (e.g., meningiomas or pituitary adenomas). Intra-axial brain tumors are particularly difficult to treat, especially at advanced stages, when they are usually discovered due to the symptoms caused by the mass effect on the surrounding brain [5]. Treatment failure can be due to several factors, including the limited capacity of current imaging modalities to identify the boundaries of the lesion within the normal appearing brain parenchyma [24]. Hence, more advanced imaging techniques for assessment of brain tumors and surrounding structures is critical to improve overall management [5,24,25]. Extra-axial brain cancers also require special attention, as these tumors (such as pituitary adenoma and meningiomas) can result in complications and long-term impairment [26,27,28].

MRI is the workhorse for brain tumor imaging in clinical practice providing structural, microstructural, functional, and metabolic information [29]. Moreover, novel advanced imaging techniques are continuously developed to improve identification, characterization, and response assessment of brain tumors [6]. Hence, many artificial intelligence (AI) applications in brain tumor imaging have been based on MRI. For more information on brain tumors, kindly refer to [5].

### 2.2. Deep Learning

**Figure 1:**
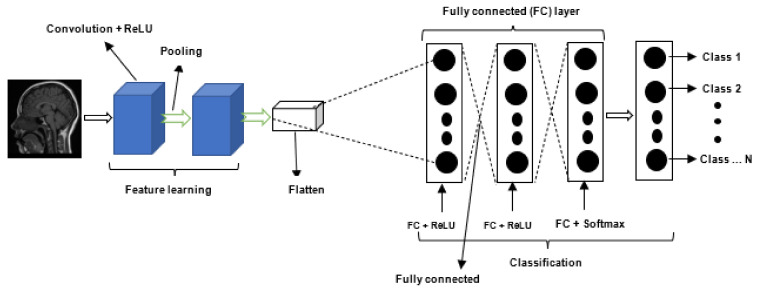
General architecture of a Convolutional Neural Network (CNN).

Deep Learning (DL) is a subfield of ML that is concerned with techniques inspired by neuroscience [30]. However, Goodfellow et al. [31] noted that neuroscience is no longer the primary source of inspiration for deep learning. Recently, DL algorithms have established themselves as a critical component of medical image analysis tasks, such as object recognition, classification, and segmentation. CNNs represent the most often utilized DL algorithm for developing brain tumor classification and segmentation techniques [3]. CNNs can learn the spatial relationships between voxels in an MRI scan. In CNNs, multiple filters are hovered on an input image with the objective of learning different features that characterize the image. A typical CNN model mainly consists of the following components: (i) input layer, (ii) convolution layer, (iii) activation function, (iv) pooling layer, (v) fully connected layer, and an (vi) output layer. The input layer is used to feed the input image into the network for processing by the successive layers. Convolution, pooling, and activation functions are used to extract high-level features from the image [3], whilst the fully connected layer is used for image classification, object segmentation, or object detection. The output layer is used to generate the network’s final prediction or results. Figure 1 illustrates the general structure of a CNN.

### 2.3. Vision Transformers

CNNs have demonstrated state-of-the-art performance in computer vision tasks, such as brain tumor segmentation and classification over the last few years. However, CNNs cannot efficiently capture long-range information or dependencies due to their small kernel size [32]. Long-range dependencies are those in which the desired output depends on image sequences presented at distant times. Due to the similarity of human organs, many visual representations in medical images are organized in sequence [33]. Destruction of these sequences will significantly affect the performance of a CNN model. This is because the dependencies between medical image sequences (such as modality, slice, and patch) contain significant information [33]. These long-range dependencies can be effectively handled by techniques that can process sequence relations. A self-attention mechanism in ViTs [20] has the capacity to model long-range dependencies which is very important for precise brain tumor segmentation. They achieve this by modeling pairwise interactions between token embeddings, thus enabling ViT-based models to learn local and global feature representations [34]. ViT has demonstrated promising performance on a variety of benchmark datasets [32,35].

**Figure 2:**
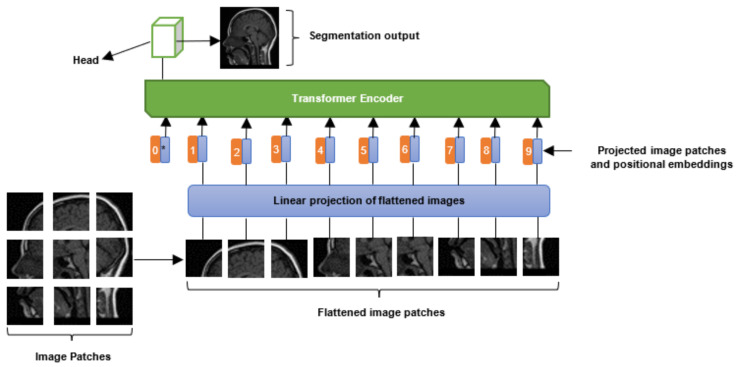
Overview of a Vision Transformers (ViT) model. The image is partitioned into *N* small patches (e.g., 9 patches). Each of the image patches contains *n* × *n* pixels (e.g., 16 × 16 pixels). After partitioning, each image patch is flattened: each of the flattened image patches is fed into a linear projection layer to obtain a lower-dimensional linear embedding. Moreover, positional embeddings are added to the sequence of image patches to ensure that each image keeps its positional information. The input sequences and position embedded sequence are fed into a standard transformer encoder for training. The training can be conducted by an MLP or CNN head stacked on top of the transformer. The “*” symbol refers to an additional learnable (class) embedding that is appended to the sequence based on the position of the image patch. This class embedding is used to predict the class of an input image after self-attention updates it.

Figure 2 depicts a high-level overview of a ViT model. As shown in the scheme, an image is partitioned into *N* small patches (e.g., 9 patches). Each of the image patches contains *n* × *n* pixels (e.g., 16 × 16 pixels). After partitioning, each image patch is flattened, such that the input sequence will consist of a flattened vector of pixel values. Moreover, each of the flattened image patches is fed into a linear projection layer to obtain a lower-dimensional linear embedding. Moreover, positional embeddings are added to the sequence of image patches to ensure that each image keeps its positional information. Finally, the input sequences and position embedded sequence are fed into a standard transformer encoder for training. The training can be conducted by a multilayer perceptron (MLP) or CNN head stacked on top of the transformer. For more information on ViT, please refer to [20].

### 2.4. Capsule Neural Networks

**Figure 3:**
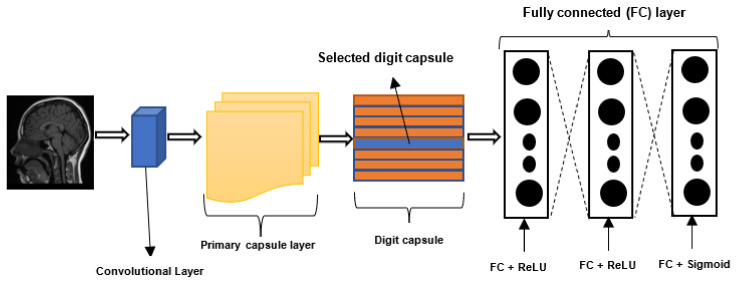
General scheme of a capsule neural network (CapsNet). A CapsNet is a three-layer network composed of convolutional, primary capsule, and class capsule layers. The primary capsule layer is typically the first one, followed by an undetermined number of capsule layers. The capsule layer is followed by the class capsule layer. The convolutional layer is used to extract features, which are then transmitted to the primary capsule layer. The primary capsule performs a series of operations and transmits the resulting feature map to the digit capsule.

Despite the remarkable success of CNNs, there are some drawbacks associated with them. First, CNNs require vast datasets for training. Second, CNNs are typically not robust to affine rotations and transformations [36]. Additionally, the routing mechanism employed by CNN’s pooling layers is distinct from that employed by the human visual system. The CNN pooling layer routes all the information extracted from the image to all the neurons in the subsequent layer, neglecting essential details or little objects in the image [37]. Hinton et al. [38] designed the CapsNet to address the drawbacks of CNN. The general structure of a CapsNet is depicted in Figure 3. A CapsNet is a three-layer network composed of convolutional, primary capsule, and class capsule layers [39]. The primary capsule layer is typically the first one, followed by an undetermined number of capsule layers. The capsule layer is followed by the class capsule layer. The convolutional layer is used to extract features, which are then transmitted to the primary capsule layer. The primary capsule performs a series of operations and transmits the resulting feature map to the digit capsule. Typically, the digit capsule is composed of a *n × m* weight matrix, where *n* denotes the number of classes and *m* the size of each digit capsule. The digit capsule is used to classify the input image before it is fed into the decoder. The decoder consists of three fully connected layers that are used to reconstruct or decode the selected digit capsule into an image.

CapsNet can recognize spatial and hierarchical relationships among objects in images [40]. They are resistant to rotation and image transformations [38]. Additionally, as shown in [41], CapsNet requires substantially less training data than CNN [18]. Moreover, results reported in the literature [41] show that CapsNet has the potential to improve the accuracy of CNN-based brain tumor diagnosis using a very small number of network parameters [42].

It is worth noting that the pooling operation in CNNs makes it robust to small input transformation. However, for CNN to perform well, it must be trained on augmented data in terms of scale, rotation, and varying perspective. Despite this, results reported in the literature [39,43] indicate that, in some cases, CapsNet performs comparably to CNN models trained on augmented datasets. CapsNet does not need to be trained on large-scale or augmented data to produce very good results. This makes it a suitable model for medical image datasets, which are typically small. For more information on CapsNet, please refer to [38].

## 3. Materials and Methods

**Figure 4:**
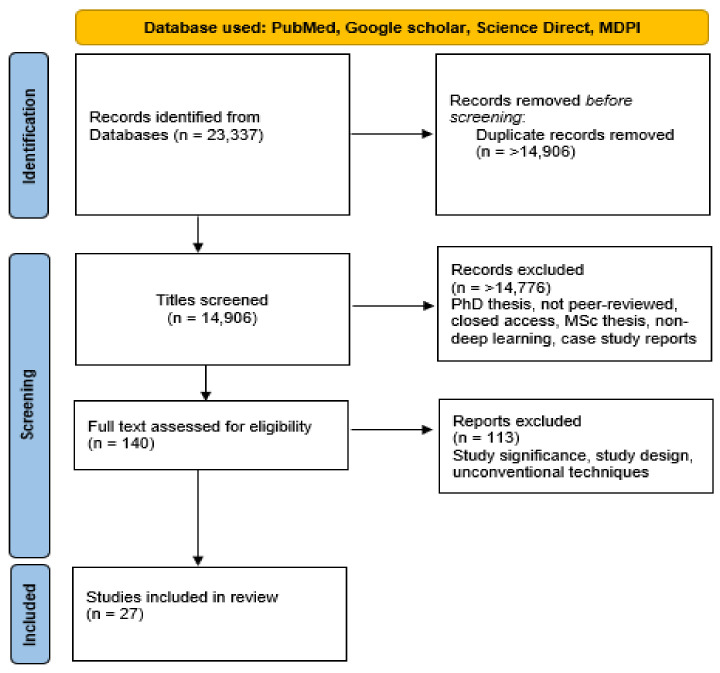
Preferred Reporting Items for Systematic Reviews and Meta-Analyses (PRISMA) diagram of the proposed review on AI applications to brain tumor MRI.

**Figure 5:**
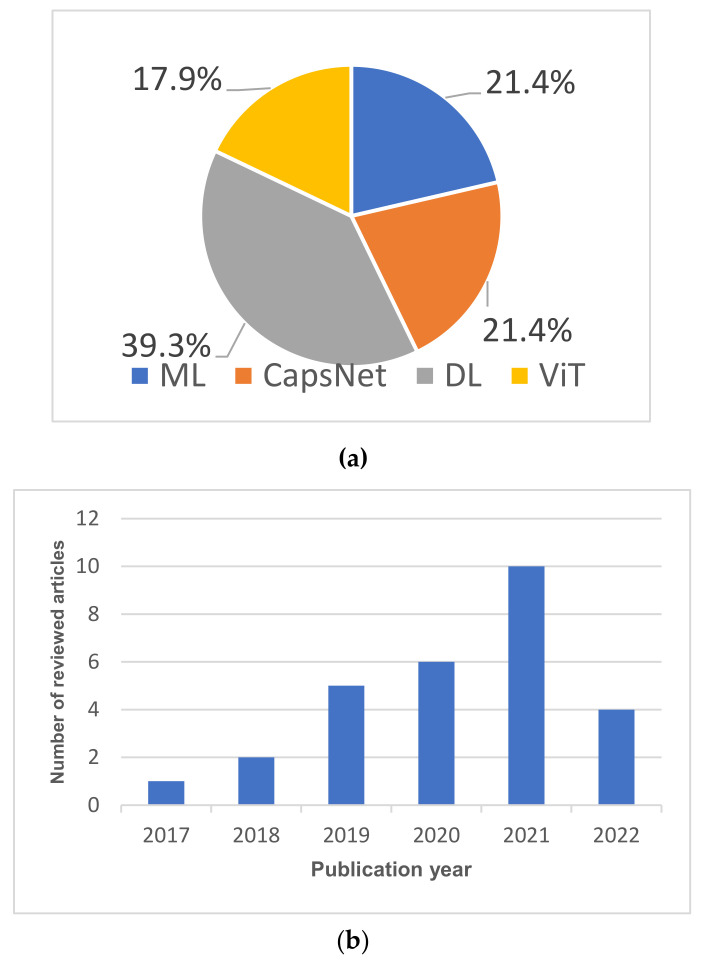
(**a**) percentage of reviewed articles; (**b**) number of reviewed articles and their publication year.

This review includes papers published between 2019 and 2022. A few studies that were published before 2019 are also covered in this paper. Specifically, we focused on papers that developed brain tumor classification and segmentation approaches using ML, CNN, CapsNet, and ViT. The following databases for scientific literature were queried to find relevant articles: PubMed, Google Scholar, and ScienceDirect. We also queried the online database of the Multidisciplinary Digital Publishing Institute (MDPI) for journal articles. The following search terms were used for our queries: brain tumor, segmentation, classification, and DL. In addition, the union of the outlined search terms was used with a set of terms relating to DL brain tumor segmentation and classification including classic machine learning, convolutional neural networks, capsule networks, and transformers. The following inclusion criteria were used in this survey: conventional brain segmentation and classification techniques, deep learning, capsule networks, vision transformers, MRI images, and peer reviewed. Ph.D. theses, M.Sc. theses, and case study papers were excluded from this study. Figure 4 shows the Preferred Reporting Items for Systematic Reviews and Meta-Analyses (PRISMA) diagram used for this survey. Figure 5a,b illustrate the percentage of articles reviewed in this study and their publication year, respectively.

### 3.1. Datasets

The Medical Image Computing and Computer Assisted Intervention (MICCAI) Society has funded numerous events and open challenges over the years to stimulate the development of DL tools and medical devices for computer-aided diagnosis. Most studies used the datasets provided by the MICCAI Society to evaluate the efficiency of their techniques. Details of the other datasets are also shown in Table 1. As shown in the table, most of the benchmark datasets are small, making it challenging to build DL models from end-to-end.

### 3.2. Image Pre-Processing Techniques

Image pre-processing techniques can be used to improve the performance of DL-based techniques. Thaha et al. [17] introduced a skull stripping and image enhancement technique for image pre-processing. Skull stripping is used to remove signals from outside the brain, removing unwanted information and, therefore, facilitating learning tasks. Image enhancement techniques are also utilized to further increase the image’s quality, allowing for the identification of essential features in the image. Sérgio et al. [60] introduced an intensity normalization technique for image pre-processing. Results obtained in the study showed that intensity normalization combined with data augmentation produced good results.

One of the key challenges encountered by researchers applying quantitative analyses to MRI scans is the presence of background interference, such as thermal noise, and/or scanner-related artifacts. Thermal noise is typically triggered by random fluctuations within the MRI system, radiofrequency coils in the MRI scanner [61], and small movements of the patient during the scanning process [62]. The presence of noise in an MR scan can reduce the quality of the image [63]. Training a CNN on noisy images can affect its ability to effectively extract tumor-related features, which will consequently affect its accuracy and generalization performance. In view of this, some studies [64,65] adopted denoising and contrast enhancement techniques [66,67] as a pre-processing step to improve the quality of MRI scans before training CNN models. Some studies also developed other techniques for reducing noise in MR images including modified median noise filter [68], Wiener filter [69], and non-local means approach [70,71]. More robust and effective denoising techniques are still required [72].

### 3.3. Performance Metrics

Several metrics were used to evaluate the performance of ML and DL techniques. Most studies [10,17,73,74] used the Dice similarity coefficient to evaluate the performance of brain tumor segmentation techniques. The coefficients determine the amount of spatial overlap between the ground truth segmentation (*X*) and the network segmentation (*Y*) [74]. Some studies used average Hausdorff Distance [73] for brain tumor segmentation. Many studies [17,19,40,62,75] used classification accuracy, precision (or recall), sensitivity, and specificity to evaluate brain tumor classification techniques. Kindly note that, when sensitivity is used along with precision, it is commonly referred to as recall.

## 4. Literature Survey

Brain tumors can be located anywhere in the human brain and assume virtually any shape, size, or contrast (dissimilarity) [10]. This shows that ML-based solutions that can effectively and automatically classify and segment brain tumors are needed. The introduction of powerful computing devices and lower hardware prices have prompted the scientific community to develop numerous techniques for brain tumor segmentation and classification. Some of the techniques were designed with classical ML algorithms, while other techniques were designed with CNN algorithms and CapsNet. This section presents a review of ML-based, CNN-based, CapsNet-based, and ViT-based techniques for brain tumor segmentation and classification. It is expected that these techniques will assist medical practitioners in improving the accuracy and consistency of diagnosis.

### 4.1. Classical Machine Learning Based Techniques

Numerous brain diagnostic systems have been developed using classical ML algorithms including Support Vector Machines (SVMs), Random Forests (RFs), k-Nearest Neighbor (k-NN), to list a few. These algorithms are used alone or in combination with other ML algorithms or feature selection techniques. This section presents a survey of ML-based brain classification and segmentation techniques.

#### 4.1.1. Brain Tumor Classification and Segmentation Using Hybrid Texture-Based Features

The texture of an image is an important feature that can be used to identify different regions of interest. The texture of a region in an image is determined by the distribution of Gray levels across the image pixels [15]. Jena et al. [15] proposed a brain tumor classification and segmentation technique using texture features and multiple ML algorithms. The technique is divided into two stages: tumor classification and tumor segmentation. In the tumor classification stage, the MRI scans are pre-processed, and texture features are extracted from the images using different texture extraction techniques. The following texture-based features were explored in the study: first-order statistical feature [76], Gray-level co-occurrence matrix (GLCM) feature [77], Gray-level run length matrix (GLRLM) feature [78], Histogram-oriented gradient (HOG) feature [79], Local binary patterns (LBP) feature [80], Cross-diagonal texture matrix (CDTM) feature [81], and simplified texture spectrum feature [82]. All the features were extracted from 100 tumor and 100 non-tumor images. The extracted features were combined to form a feature vector matrix of size 200 × 471. Subsequently, the feature vector matrix was used to train five ML algorithms: SVM, k-NN, binary decision trees, RF, and ensemble methods. The ensemble methods consist of seven algorithms, namely: Adaboost, Gentleboost, Logitboost, LPboost, Robustboost, RUSboost, and Totalboost. After training, the tumorous images were identified and used as input to a hybrid tumor segmentation technique designed in the study. The hybrid technique consists of k-NN and fuzzy C-means clustering algorithms. The hybrid technique was used to segment the tumor regions in the images, and it was evaluated on two datasets based on the following performance metrics: average Dice similarity coefficient (DSC), average Jaccard similarity coefficient, and average accuracy. The dataset used to evaluate the model include: BraTS2017, BraTS2019, and the Cancer Imaging Archive (TCIA). Experiments show that the ensemble methods produced the best result, achieving a classification accuracy of 96.98% and 97.01% for BraTS2017 + TCIA and BraTS2019 + TCIA, respectively. RF produced the second-best result, achieving an accuracy of 96.5% and 96.99% for BraTS2017 + TCIA and BraTS2019 + TCIA, respectively. The results also show that the segmentation technique produced a Dice similarity score and accuracy of 90.16% and 98.4%, respectively for BraTS2017.

#### 4.1.2. Brain Tumor Classification Using GoogleNet Features and ML

Sekhar et al. [14] proposed a tumor classification model using a modified GoogleNet pre-trained CNN model [83] and two ML algorithms: SVM and k-NN. In the study, the last three fully connected layers of GoogleNet network were modified and fine-tuned on brain tumor images. After fine-tuning, the 1024 feature vector from the last average pooling layer was extracted and used to train SVM and k-NN classifiers. The technique was evaluated on the CE-MRI dataset [56] containing 3064 T_1_w post GBCA brain MR images from 233 patients. Experimental results show that GoogleNet produced precision and recall of 96.02% and 97.00% for glioma, respectively, using softmax activation function. The performance of the model was improved by over 2.5% when the SVM classifier was used. It achieved precision and specificity of 98.76% and 98.93% for glioma, respectively. The performance of GoogleNet was also improved by over 2.3% when the k-NN classifier was used. It produced precision and specificity of 98.41% and 98.63% for glioma, respectively. This shows that features extracted from pre-trained CNN models can be used to build effective ML-based classifiers.

#### 4.1.3. Brain Tumor Classification Using Ensemble of Deep Features and ML Classifiers

Kang et al. [84] proposed a method for brain tumor classification using an ensemble of deep features. The technique consists of three stages. In the first stage, input images are pre-processed, and more images are generated using data augmentation. The pre-processed images are then used as input to 13 pre-trained CNN models, namely: ResNet-50 [85], ResNet101 [85], DenseNet-121 [86], DenseNet-169 [86], VGG-16 [87], VGG-19 [87], AlexNet [88], Inception-v3 [89], ResNeXt-50 [90], ResNeXt-101 [90], ShuffleNet-v2 [91], MobileNet-v2 [92], and MnasNet [93]. The pre-trained CNN models are used to extract features from the images. In particular, the features are extracted from the fully connected layers of the pre-trained models. The extracted features are used to train nine ML classifiers, namely: Gaussian Naïve Bayes, Extreme Learning Machine (ELM), Adaptive Boosting (AdaBoost), k-NN, RF, SVM and neural networks with a fully connected layer. Moreover, the pre-trained models that produced the three best results are identified, and the extracted features from the three best pre-trained models are combined into one sequence. Finally, the combined features are used to train the nine ML classifiers. The technique was evaluated on three brain MRI datasets downloaded from Kaggle websites. Results showed that DenseNet-169, Inception-v3, and ResNeXt-50 produced the best features, achieving an accuracy of 96.08%, 92.16%, and 94.12%, respectively.

#### 4.1.4. Brain Tumor Detection Using Metaheuristics and Machine Learning

Kaur et al. [94] introduced a brain tumor classification technique using multiple metaheuristic and ML algorithms. In the study, brain MRI was pre-processed, and features were extracted from the images using Principal Component Analysis (PCA) and Independent Component Analysis (ICA). The extracted features were further reduced by using different optimization algorithms, including cuckoo search, lion optimization, and bat optimization algorithms. Furthermore, the optimized dataset was used to train two ML algorithms: NB algorithm and Residual Neural Network (ResNet50). Three case studies were considered in the study. In the first case study, PCA was used for feature selection, the firefly algorithm was used for image smoothing, and the naïve Bayes (NB) classifier was used for image classification. In the second case study, ICA was used for feature extraction, the cuckoo search algorithm was used for image smoothing, and the NB classifier was used for tumor classification. In the third case study, ICA was used for feature extraction, the combination of lion and bat optimization algorithms was used for image smoothing, and RNN was used for tumor classification. Experiments performed on the Cancer Imaging Archive (TCIA) dataset show that PCA + firefly algorithm + NB algorithm outperform ICA + cuckoo search + NB algorithm, achieving an accuracy of 96.59%. The results also show that ICA + lion optimization + bat optimization + RNN produced the best accuracy of 98.61%.

#### 4.1.5. Categorization of Brain Tumor Using CNN-Based Features and SVM

Deepak and Ameer [13] proposed an automated system for brain tumor categorization using SVM and CNN. In the study, a CNN was used to extract image features from MRI images. The CNN consists of five convolutional layers and two fully connected layers. The feature maps from the fifth convolution layer and the 1st fully connected layer are extracted and used separately to train SVM for multiclass classification. The activations of the fifth convolution layer contain 3136 feature vectors, while the activations of the 1st fully connected layer contain 10 feature vectors. No feature selection algorithm was used in the study. The technique was tested on the FigShare dataset. The dataset consists of 3064 T_1_w post GBCA MR images (from 233 patients) belonging to three classes of brain tumors: glioma, meningioma, and pituitary tumor. The proposed technique achieved an accuracy of 95.82% when trained on the 10 feature vectors extracted from the fully connected layer. The accuracy reduced to 93.83% when it was trained on the 3136-feature set extracted from the fifth convolution layer. This shows that SVM models trained on small-scale feature sets have the potential to produce better results than those trained on large-scale feature sets. CNN produced an accuracy of 94.26% when it was trained as a standalone classifier.

**Figure 6:**
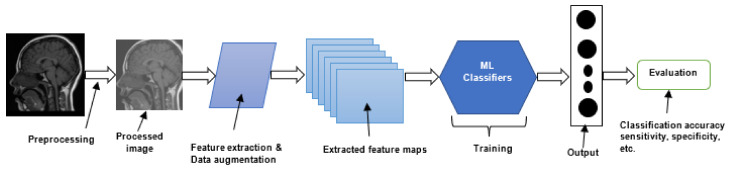
Building blocks of a typical ML-based brain tumor classification and segmentation model.

The summary of all the ML-based algorithms is presented in Table 2. Furthermore, the building blocks of a typical ML-based brain tumor segmentation and classification model are shown in Figure 6.

### 4.2. Deep Learning-Based Brain Tumor Classification Techniques

This section discusses some of the CNN-based brain tumor classification techniques that have been developed in the literature.

#### 4.2.1. Brain Tumor Multi-Grade Classification

Sajjad et al. [16] proposed a CNN-based multi-grading technique for brain tumor classification. The technique is divided into three stages: brain tumor segmentation, data augmentation, and fine-tuning. In the first stage, the authors used a CNN-based architecture (called InputCascadeCNN [10]) to segment brain tumors from the MRI images. The architecture consists of 7 × 7 feature maps for extracting local features (small details of the tumors) and 11 × 11 feature maps for extracting global features. In the second stage, the authors applied four different data augmentation techniques to the segmented brain tumors for geometric transformation invariance, including flipping, rotation, skewness, and shears. The authors also applied another set of four data augmentation techniques for noise invariance: sharpening, Gaussian blur, emboss, and edge detection. Finally, in the third stage, the authors used the VGG-19 architecture to fine-tune the augmented data. The technique was evaluated on two datasets: Radiopedia [55] and a brain tumor dataset collected by Cheng et al. [56]. For the Radiopedia dataset, before data augmentation, the technique produced an accuracy of 90.03%, 89.91%, 84.11%, and 85.50% for grades I, II, III, and IV, respectively. After data augmentation, the accuracy for the four grades increased to 95.5%, 92.66%, 87.77%, and 86.71%. In addition, for the brain tumor dataset, the technique produced a sensitivity and specificity of 84.51% and 93.34%, respectively, before data augmentation. The sensitivity and specificity increased after data augmentation to 88.41% and 96.12%, respectively. This underscores the usefulness of data augmentation in improving the accuracy and generalization performance of CNN models.

#### 4.2.2. MR Brain Image Classification Using Differential Feature Maps

Isselmou et al. [95] proposed a CNN technique for MR brain image classification using differential deep CNNs. In traditional CNNs, normal feature maps are created using random initialization or transfer learning. However, in this study [95], differential feature maps were produced by applying user-defined hyperactive values and a differential operator introduced by Lei et al. [96]. The produced differential convolution maps are used to analyze the directional patterns of voxels and their neighborhoods by calculating the difference between pixel activations. Readers can learn more about differential feature maps from [95]. The technique was evaluated on 17,600 normal and abnormal MR images collected from Tianjin Universal Center of Medical Imaging and Diagnostic (TUCMD). Several data augmentation techniques were proposed in the study to improve the generalization performance of the classification model. The data augmentation increased the size of the dataset to 25,000. Results show that the differential feature maps improved the performance of the model. The results also show that the proposed technique can classify many MR images with high accuracy. The technique achieved a classification accuracy, sensitivity, and specificity of 99.25%, 95.89%, and 93.75%, respectively.

#### 4.2.3. Brain Tumor Classification for Multi-Class Brain Tumor Image Using Block-Wise Fine-Tuning

Swati et al. [97] introduced a block-wise fine-tuning technique for multi-class brain tumor MR images. They considered three classes of abnormal brain tumors: (i) glioma, (ii) meningioma, and (iii) pituitary tumors. In the study, the VGG-19 pre-trained network was used to initialize the weights of the CNN model designed in the study. Additionally, rather than fine-tuning the VGG-19 pre-trained network all at once, the previous layers of the CNN were fine-tuned using a block-wise approach. In this approach, the 19 layers of the VGG-19 architecture were divided into six blocks. The first and second blocks consist of two convolutional layers and one pooling layer. The third, fourth, and fifth blocks consist of four convolutional layers and one pooling layer. The last block consists of two fully connected layers and one output layer. The block-wise fine-tuning is initiated by fine-tuning the layers in the last block and freezing the weights of the layers in the other blocks. A similar approach is taken until the whole six blocks were fine-tuned. The block-wise fine-tuning technique was evaluated on the CE-MRI dataset [56]. Results showed that the technique achieved a classification accuracy of 94.82% using the 5-fold cross-validation.

#### 4.2.4. Multi-Scale 3D CNN for MRI Brain Tumor Grade Classification

Many of the two-dimensional (2D) CNN models do not totally learn volumetric information in MR images. They are most capable of extracting features from 2D slices. Mzoughi et al. [62] tackled this problem by developing a multi-scale three-dimensional (3D) CNN architecture for grading MRI brain tumors into LGG and HGG. The proposed architecture consists of eight 3D convolutional layers, three fully connected layers, and small 3D kernels at each convolutional layer. The 3D convolutional layers provide a detailed feature map that can explore the volumetric information on MRI [62]. The 3D feature map also learns both local and global features with high classifying power. The small 3D kernels (3 × 3) were used to improve the computational complexity of the network and reduce the number of weights in the network. Additionally, the authors [62] developed a technique for removing thermal noise and distortions generated by the magnetic field and minor movements of the patients during the scanning procedure. The technique is based on intensity normalization [60] and adaptive contrast enhancement [98]. Data augmentation was also used in the study to generate more images and improve the generalization performance of the model. The technique was evaluated on the BraTS2018 dataset, and it produced a classification accuracy of 96.49% with data augmentation and 82.4% without data augmentation. The results also show that max-pooling outperforms average pooling and small kernel size performs better than large kernel size for brain tumor grade classification. This shows that data augmentation, small kernel size, and max-pooling play a role in improving the performance of the 3D brain tumor grading model.

#### 4.2.5. Brain Tumor Classification Using Pairwise Generative Adversarial Networks

Ge et al. [75] tackled the problem of small-scale datasets using augmented brain MR images. They used a pairwise generative adversarial network (GAN) to generate synthetic MR images for four types of MRI’s techniques, namely: T_1_w, GBCA, T_1_w (T_1_w post GBCA), T_2_w, and T_2_ fluid-attenuated inversion recovery (FLAIR). In the study, 2D MRI slices were extracted from the three views of 3D volume images: coronal, axial, and sagittal. The extraction is performed for each of the four modalities: T_1_w, T_1_w post GBCA, T_2_w, and FLAIR. The extracted 2D MRI slices were divided into training, validation, and testing subsets. Furthermore, a pairwise GAN model was used to generate synthetic MRI for the training subset. The pairwise GAN used a pair of inputs in two streams. It is designed to handle the following two scenarios: (a) generate synthetic images of fake patients with the objective of enlarging the training subset; (b) generate synthetic images for patients with missing modalities of MRI. The synthetic images are generated from another modality of MRI for the same patient to replace the missing modality. A two-stage training technique was introduced in the study based on the findings that the distributions of synthetic images vary from the distribution of real images. The two-stage training is carried out in the following manner: firstly, the entire network was trained on the augmented images for glioma subtype classification. Afterwards, the real MR images are used to refine the network. The U-Net architecture was adopted in the study. The final output of the architecture is the glioma class for each slice in an MR image. In view of this, for each patient, the subtype for each MRI slice was taken into consideration, and the final diagnosis or subtype classification for the patient will be made based on a majority vote. The network was evaluated on a dataset containing 3D brain volume images obtained from the Cancer Genome Atlas Glioblastoma Multiforme (TCGA-GBM) collection [99] and the Cancer Genome Atlas Low Grade Glioma (TCGA-LGG) [99] collection using 5-fold cross-validation. Different case studies were considered in the experiments, and the case study that produced the best result achieved an average classification accuracy, sensitivity, and specificity of 88.82%, 81.81%, and 92.17%, respectively.

**Figure 7:**
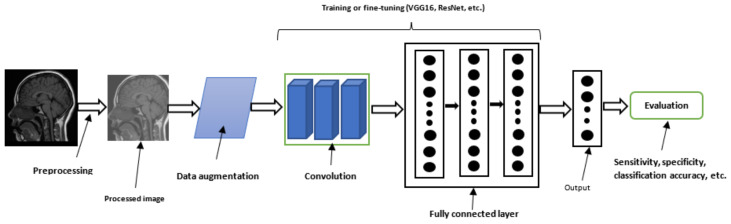
Workflow of deep learning (CNN)-based brain tumor classification techniques.

The flow for most of the proposed CNN-based brain tumor classification technique is shown in Figure 7. The summary of all the CNN-based brain classification algorithms is presented in Table 3.

### 4.3. Deep Learning-Based Brain Tumor Segmentation Techniques

This section summarizes some of the CNN-based brain tumor segmentation techniques that have been published in the literature.

#### 4.3.1. Enhanced CNN and Bat Algorithm for Brain Tumor Classification

Thaha et al. [17] introduced a strategy for segmenting brain tumors using an enhanced CNN coupled with the Bat algorithm. The Bat algorithm was used to optimize the loss function (cross-entropy). The loss function is adjusted with the goal of improving the segmentation accuracy. In the experiments, MR images were preprocessed using a skull stripping and image enhancement technique. The authors added a central-point-enhanced layer in the study to address misclassification concerns. The approach was evaluated, and the results indicated that the improved CNN achieved a precision, recall (or sensitivity), and accuracy of 87%, 90%, and 92%, respectively, compared to the standard CNN, which achieved 82%, 85%, and 89%, respectively.

#### 4.3.2. Encoder–Decoder Architecture for Brain Tumor Segmentation

Zeineldin et al. [41] introduced a CNN architecture (called deepSeg) for detection and segmentation of brain tumors using MRI. The architecture consists of two segments, namely: a contracting path (or encoder) and an expansive path (decoder). The encoder is composed of two 3 × 3 convolutional layers, each followed by a pooling and rectified linear unit (ReLU). The feature map from the encoder unit is passed to the decoder, which upsamples the feature maps. The decoder consists of deconvolution layers, one 2 × 2 up-convolution layer, one concatenation layer, two 3 × 3 convolution layers, and one ReLU. Data augmentation was used in the study to improve the model’s performance. Batch normalization was introduced between each convolution and ReLU layer to enable each layer to learn features independently from other layers. The technique was evaluated on the BraTS2019 dataset. The dataset was obtained from different scanners, protocols, and 19 institutions. In view of this, the training images may be noisy. Therefore, the N3 bias correction tool [100] was used in the study to normalize the image vectors and correct the bias. Results from the experiments show that the proposed technique produced a DSC, sensitivity, and specificity of 0.814, 0.783, and 0.999, respectively.

#### 4.3.3. Patching-Based Technique for Tumor Segmentation

AlBadawy et al. [73] proposed a patching-based technique for brain tumor segmentation using CNNs. They assessed the impact of CNN training on datasets from various institutions. Forty-four GBM patients from two centers were considered to construct three CNN models. The first CNN was trained using data from patients at the first institution, whereas the second CNN used data from patients at the second institution. The last CNN was trained on patients’ data from the two institutions. Each CNN was composed of four convolution layers and two fully connected layers. Patches of equal size were extracted from the dataset and grouped into three classes, namely: tumor patches, healthy patches near the tumor, and other healthy patches. Based on patient data, the authors further categorized the tumor images into six classes: 0 (normal), 1 (ground truth region based on a combination of classes 2–5), 2 (enhancing region), 3 (necrotic region), 4 (T1-abnormality), and 5 (T2-abnormality). The models were evaluated using DSC and the Hausdorff distance. The performance metric was utilized to compare the ground truth with the automatic segmentation. The performance of several techniques was compared using 10-fold cross-validation method. The results demonstrated that CNN models achieved the following Dice coefficients when it was evaluated on different institutional data: 0.68 ± 0.19 and 0.59 ± 0.19, respectively. The performance declined when CNN was trained on the same institutional data. It produced a Dice coefficient of 0.72 ± 0.17 and 0.76 ± 0.12, respectively. The authors stated that further research is necessary to determine the cause of this performance decrease.

#### 4.3.4. Two-Path or Cascade Architecture for Brain Tumor Segmentation

Havaei et al. [10] introduced a DNN technique for segmenting LGG and HGG from MRI scans. The network was designed to simultaneously learn both local and global features. A two-pathway architecture comprised of two streams was proposed to accomplish this task. The two-pathway architecture was introduced so that two factors will impact the prediction of a pixel’s label: the visual details of the area surrounding the pixel and its larger context, or the patch’s position in the brain. The first pathway consists of 7 × 7 convolutional filters that are used to segment localized or minute aspects of the tumor (focuses on small details of the tumor at the local scale or local detailed feature). The second pathway utilizes larger convolution filters 13 × 13 to learn larger details of the brain tumor (it focuses on global contextual features). The segmented image is created by concatenating the output (or feature maps) from the two pathways. The concatenated feature map is then fed into the output layer.

Havaei et al. [10] noted that one disadvantage of the two-pathway network is that it predicts each segmentation label independently of the others, in contrast to the majority of segmentation methods which introduce joint segmentation models that account for the direct dependencies between spatially adjacent labels. Typically, these types of approaches require more computation than a simple feed-forward approach. Havaei et al. [10] addressed this issue by developing a cascade architecture. The architecture investigates the efficiency of CNNs and models the segmentation dependencies between adjacent labels. The cascade architecture consists of two CNNs. The first CNN’s output probabilities are used as an additional input to the second CNN’s layers. The output of the first CNN is simply concatenated with any of the layers of the second CNNs in this scenario. The authors [10] noted that the model’s belief about the value of nearby labels had an effect on the network’s final prediction.

In the study, Havaei et al. [10] examined three cascade architectures. In the first cascade architecture, the output of the first CNN is directly concatenated with the input of the second CNN. The output is treated as a second image channel in the patch’s input. In the second cascade architecture, the output of the first CNN is concatenated with the output of the first hidden layer in the second CNN. In the third architecture, the output of the first CNN is concatenated with the output of the second hidden layer of the second CNN. The three architectures were tested on an unbalanced dataset. In addition, 98% of the total labels are healthy, while the remaining are pathological voxels: necrosis (0.18%), edema (1.1%), non-enhancing tumor (0.12%), and enhancing tumor (0.38%). Overfitting will occur if a network is trained on this skewed dataset. As a result, the authors developed a two-stage training technique. The authors began by creating a dataset (dubbed the patches dataset) in which all labels had the same probability. On the constructed dataset, the networks were trained. The second training phase involved retraining the network’s output layer with a more representative label distribution. The previous layers of the pre-trained network were fixed during re-training. The preceding layers account for class diversity, while the output layer takes care of calibrating the output probabilities (thanks to the re-training that was performed on the output layer).

The un-cascaded and cascaded models were subjected to a variety of experiments. Results from the un-cascaded models indicated that the double-pathway models generated the best results. The results also indicated that adding a second training phase considerably improved the model’s performance. Additionally, training the local and global routes concurrently produced better outcomes than training each pathway separately and averaging the results. The results from the cascaded models showed that the first cascade architecture produced the best Dice similarity of 0.88. The models increased the Dice similarity scores for all tumor regions.

#### 4.3.5. Triple CNN Architecture for Brain Tumor Segmentation

Training three separate networks for different binary segmentation tasks is less time-consuming and computationally demanding than training a single network for multiclass segmentation, which is more time-consuming and computationally demanding. On this basis, Yogananda et al. [74] developed a triple network architecture for brain segmentation based on CNN. Three distinct network topologies were created in the experiment to simplify the multiclass segmentation problem to a single binary segmentation problem for each of the three networks. Each model was trained independently to perform a binary task of predicting Whole Tumor (WT), Tumor Core (TC), and Enhanced Tumor (ET). The output of the three networks is combined using a triple volume fusion to create a segmentation volume with multiple classes. The input images (T_1_w, T_2_w, FLAIR, and T_1_w post GBCA) were processed through a single initial convolution layer for each model. The convolution layer generates 64 feature maps, which are then used to form seven dense blocks. Each dense block is composed of five interconnected layers. Each of the five layers contains four sublayers that are connected sequentially. These sublayers include batch normalization, ReLU, three-dimensional convolution, and three-dimensional spatial dropout. Each layer’s input was utilized to build different feature maps, which were then concatenated with the next layer’s input. Numerous studies were conducted to determine the network’s efficacy. In addition, 3-fold cross-validation was performed on the BraT2018 dataset to increase the network’s generalization performance. Using 75% overlapping patches, the technique produced an average DSC of 0.90, 0.82, and 0.79 for WT, TC, and ET, respectively. Additionally, it produced a DSC of 0.92, 0.84, and 0.80 for WT, TC, and ET, respectively, when 85% of the patches overlapped.

#### 4.3.6. Brain Tumor Classification and Segmentation Using a Combination of YOLOv2 and CNN

Sharif et al. [101] proposed a framework for brain tumor analysis using MR images. The framework is divided into four stages: tumor enhancement, feature extraction and selection, localization, and tumor segmentation. In the first stage, a homomorphic wavelet filter was used to remove noise from the brain MRI. Afterwards, the processed images were transferred to an inception-v3 pre-trained model [89] for feature extraction. The features are extracted from the fully connected layers of the pre-trained model. The extracted feature vectors are also passed to the non-dominated sorted genetic algorithm (NSGA) [102] for feature selection. Furthermore, the optimized features are transferred to different classical ML algorithms for classification. The classifiers considered in the study are decision trees, Classification and Regression Tree (CART), linear discriminant analysis (LDA), SVM, k-NN, and softmax. After classification, the identified tumors were transferred to inception-v3 for feature extraction. The features are extracted from the depth concatenation (mixed-5) layer of inception-v3. The extracted features are then transferred to YOLOv2 for localization. The localized features are finally transferred to McCulloch’s Kapur entropy [103] for 3D segmentation of the brain tumor. The proposed framework was evaluated on five ML algorithms and three benchmark datasets: BraTS2018, BraTS2019, and BraTS2020. Experiments show that SVM produced the best classification accuracy of 98%, 99%, and 99% for BraTS2018, BraTS2019, and BraTS2020, respectively. The results also show that the YOLOv2-inception-v3 model produced a mean average precision (mAP) of 0.98, 0.99, and 1.00 for BraTS2018, BraTS2019, and BraTS2020, respectively.

**Figure 8:**
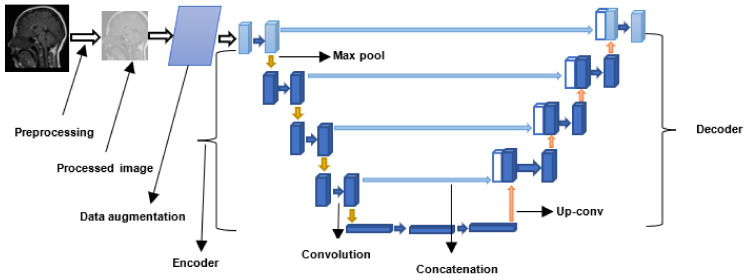
Workflow for CNN-based brain tumor segmentation.

The general workflow for CNN-based brain tumor segmentation is shown in Figure 8. Moreover, Table 4 shows the summary of all the CNN-based brain tumor segmentation techniques surveyed in this study.

### 4.4. Vision Transformers for Brain Tumor Segmentation and Classification

This section presents some recent transformer-based brain tumor classification and segmentation techniques.

#### 4.4.1. Brain Tumor Segmentation using Bi Transformer U-Net

Jia et al. [104] proposed a network for segmenting brain tumors based on CNNs and transformers. The network is composed of a decoder and a three-dimensional CNN encoder with an attention module. In the study, the input MRI scans are fed into a stack of convolutional layers. The resulting feature maps were fed into a 3 × 3 × 3 convolutional block to increase the channel dimension of the feature maps from *K* to *d*. The spatial and depth dimensions of the convolutional block’s feature map are flattened to a single dimension of size *N*. This partitions the feature map into d-dimensional tokens of size *N*. In addition, positional embedding is added to the flattened feature map. Following feature embedding, the tokenized feature representation and position embedded sequence is fed into a transformer block. The block is composed of multiple transformer layers. The output of the transformer layers is reshaped and passed through another 3 × 3 × 3 convolutional block to reduce the channel dimension from *d* to *K*. Additionally, five 3D CNN layers are used to upsample the reduced feature maps. Moreover, skip connections are introduced into the network by concatenating the outputs of the first three 3D CNN downsampling layers with the inputs of the final three upsampling layers. The technique was evaluated on the BraTS2021 dataset, and it obtained an average DSC of 0.823, 0.908, and 0.839 for ET, WT, and TC, respectively.

#### 4.4.2. Multi-Modal Brain Tumor Segmentation Using Encoder–Transformer–Decoder Structure

Developing a neural network capable of learning global dependencies is critical for segmenting brain tumors. Transformer-based networks can be used to model both the local features of images and their long-range dependencies. Wang et al. [35] designed a ViT-based network for segmenting three-dimensional brain tumors. The network is composed of two primary components: a three-dimensional CNN and transformers. The 3D CNN is made up of a 3D encoder and a 3D decoder. The encoder is used to extract volumetric features from three-dimensional brain tumor images. Additionally, the 3D CNN encoder is utilized to downsample the brain tumor images to efficiently collect the images’ local 3D features. The feature map from the encoder was fed into the transformer to capture the images’ global features. Furthermore, the resultant feature embeddings are passed into the 3D decoder, which upsamples them and generates the segmented image. The technique was evaluated on the BraTS2019 and BraTS2020 datasets. It produced a DSC of 90% for WT, 78.93% for ET, and 81.84% for TC in BraTS2019. In addition, it achieved a DSC of 90.09%, 78.73%, and 81.73% for WT, ET, and TC, respectively, for the BraTS2020 dataset.

#### 4.4.3. Brain Tumor Segmentation Using Transformers Encoders and CNN Decoder

Some of the ViT-based techniques used CNN for encoding. Ali et al. [105] introduced a ViT-based technique for 3D image segmentation that uses ViT for encoding. The transformer encoder directly accepts a one-dimensional sequence of 3D image patches as inputs. The image sequence was created by dividing the input images into non-overlapping patches of uniform size. For each layer in the transformer model, the extracted patches are projected into an N-dimensional embedding space. Moreover, a one-dimensional positional embedding was added to the embedding space to preserve the spatial information of the extracted patches. Additionally, the output was routed through a stack of transformer blocks for encoding. During the encoding, sequence representations at different resolutions are extracted from the transformer and reshaped into a tensor of a specific size. Convolutional and normalization layers are used to project the reshaped tensors from the embedding space into the input space at each image resolution. The output of the transformer’s final layer is fed into a CNN decoder. The CNN decoder employs deconvolutional layers to increase the resolution of the feature maps by a user-defined factor. All of the transformer blocks’ feature maps are concatenated and fed into another set of convolutional layers. The feature map from the convolutional layers is fed into a deconvolutional layer for upsampling. This procedure is repeated for all the layers. The final output is fed into a 3D convolutional layer with a softmax activation function to construct the predicted segmented image. On the Medical Segmentation Decathlon (MSD) dataset [59], the technique produced a DSC of 0.789, 0.585, and 0.761 for WT, ET, and TC, respectively.

#### 4.4.4. Brain Tumor Segmentation Using Swin Transformers

Hatamizadeh et al. [32] developed a technique for segmenting brain tumors (Swin UNEt TRransformers (UNETR)) based on Swin transformers developed by Liu et al. [106]. Swin transformer (Swin stands for Shifted window) is a hierarchical transformer that computes its representation using shifted windows [78]. The shifted window improves the performance of a model by restricting self-attention computation to non-overlapping windows while permitting cross-window communication [106]. The input to Swin UNETR model is a sequence of 3D image tokens with a specific dimension. Each of the 3D tokens was evenly partitioned into distinct and non-overlapping regions at each layer of the transformer. The non-overlapping windows ensure that token interactions are modeled efficiently. The window region for each layer is shifted by a specific number of voxels using a shifting mechanism described in [78]. Following that, the partitioned 3D tokens are sent to the Swin UNETR encoder. The encoder is composed of four stages, each having two transformer blocks. Throughout the four stages, a linear embedding layer is employed to generate 3D tokens of varying sizes. Additionally, a patch merging layer is employed to lower the feature representation’s resolution. Moreover, a patch merging layer is utilized to organize and concatenate several patches. A linear layer is used to further reduce the resulting feature map. The feature map is reshaped and fed into a two-layer convolutional residual block. The resultant feature map is fed into the Swin UNETR decoder. The decoder uses deconvolutional layers to boost the resolution of the feature map. The decoder outputs from the four stages are concatenated and fed into another residual block. The final tumor segmentation is computed using a convolutional layer and a sigmoid activation function. The technique was evaluated on the BraTS2021 dataset using 5-fold cross-validation. It achieved an average DSC of 0.891, 0.933, and 0.917 for ET, WT, and TC, respectively.

#### 4.4.5. Convolution-Free 3D Brain Tumor Segmentation Using Vision Transformers

The majority of transformer-based techniques were designed to accept patches of two-dimensional or three-dimensional slices. Peiris et al. [107] developed the VT-UNet transformer architecture, which is capable of directly processing 3D volumetric data for semantic segmentation. The architecture is composed of one encoder and decoder module. The encoder module is composed of three layers: a three-dimensional patch partitioning layer, a linear embedding layer, a three-dimensional patch merging layer, and two transformer encoder blocks. The 3D patch partitioning layer is used to divide the three-dimensional input volume into non-overlapping three-dimensional patches. The authors proposed two techniques for partitioning volumetric data into non-overlapping patches. The partitioned 3D tokens are fed into the linear embedding layer, which converts each token to a vector space of N dimensions. Additionally, the 3D patch merging layer is used to generate feature representation hierarchies. The encoder module’s output is passed to the decoder module. The module consists of a transformer encoder block, a 3D patch expanding layer, and a classifier. The decoder block is used to compute cross and self-attention independently. Self-attention is used to exploit global interactions between encoder features, whereas cross-attention is used to filter out local or non-semantic features. The 3D patch expanding layer is used to transform the encoded feature maps into a format compatible with the input image’s spatial resolution. The classifier layers are composed of a single 3D convolutional layer that is used to map the feature representations to the specified segmentation classes. The technique was evaluated on the BraTS2021 dataset, and it achieved a DSC of 85.59%, 87.41%, and 91.20% for ET, TC, and WT, respectively.

**Figure 9:**
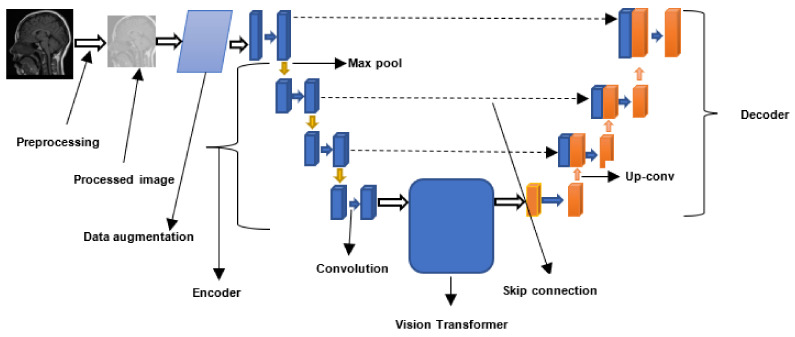
General workflow for ViT-based brain tumor segmentation techniques.

The workflow for a ViT-based brain tumor segmentation technique is shown in Figure 9. Furthermore, the summary for the ViT-based techniques reviewed in this study is shown in Table 5.

### 4.5. Capsule Neural Network-Based Brain Tumor Classification and Segmentation

Many studies used CNNs for brain tumor segmentation and classification. Very few studies explored CapsNet. This section presents some of the recent CapsNet-based brain tumor segmentation and classification techniques developed in the literature.

#### 4.5.1. Brain Tumor Classification Using Capsule Neural Network

Afshar et al. [18] designed a brain tumor classification technique using CapsNet. They explored different architectures of CapsNet with the goal of identifying the architecture that will produce the best classification accuracy. In the study, they investigated the effects of input data on CapsNet by training them on different inputs: the (i) whole brain image, and (ii) the segmented tumor region. They also adopted an early stopping approach [31] to handle the overfitting problems of CapsNet. Finally, the authors developed a visualization paradigm for the capsule network’s output to further explain the features that the model learned from the input. The authors designed and compared the performance of the following capsule network architectures: (i) two convolutional layers with 64 feature maps each, (ii) one convolutional layer with 64 feature maps, (iii) one convolutional layer + 64 feature maps and 16 primary capsules, (iv) one convolutional layer + 64 feature maps and 32 primary capsules containing four dimensions, (v) three fully connected layers containing 1024, 2048, and 4096 neurons, respectively. Experimental results show that the capsule network with one convolutional layer and 64 feature maps produced the best predication accuracy of 86.56%. The results also revealed that CapsNet performed better when trained on segmented tumors than when trained on the entire brain image. The authors also compared the performance of CNNs and CapsNets, and the results show that CapsNet outperform CNN by 14.43%. CNN and CapsNet produced a classification accuracy of 72.13% and 86.56%, respectively.

#### 4.5.2. Brain Tumor Classification Using Bayesian Theory and Capsule Neural Network

CapsNet, like other standard DL networks, suffers from model uncertainty [108]. Model uncertainty refers to the degree to which a model is uncertain about its weights and predictions. Developing a network that models uncertainty is important because it serves as a medium for returning the uncertain predictions to experts for further verification. Most CNN networks use the softmax activation function. However, the output of the softmax does not reflect the degree of uncertainty of a model’s prediction [108]. Afshar et al. [40] proposed a brain tumor classification network (called BayesCap) using Bayesian theory and CapsNet. Bayesian theory is used to model the uncertainty associated with predictions of the model. The technique was evaluated on the CR-MRI dataset [56] consisting of 3604 brain MR images from 233 patients. The results show that the technique produced an accuracy of 68.3%. The same authors [40] in another study [18] evaluated CapsNet on the same dataset, and the results show that CapsNet (without Bayesian theory) outperforms the Bayesian variant, producing a classification accuracy of 78%. The reduced accuracy was expected because the Bayesian variant was developed with the objective of modeling the uncertainty and learning the posterior of the model’s weights rather than improving the classification accuracy [40]. Overall, the results show that Bayesian theory improves the interpretability of a network, which is very important for medical applications.

#### 4.5.3. CapsNet-Based Brain Tumor Classification Using an Improved Activation Function

The ReLU activation function is popularly used in most neural network-based algorithms, including CNN and CapsNet. However, ReLU does not activate a neuron if its derivative is zero. Furthermore, the performance of a ReLU-based CapsNet brain tumor classification system requires improvement [19]. Therefore, Adu et al. [19] proposed a new activation function called parametric scaled hyperbolic tangent (PSTanh), for improved brain tumor classification. Adu et al. [19] noted that the proposed activation function facilitates faster optimization and improves the standard hyperbolic tangent by avoiding the vanishing gradient problem. In the study, a CapsNet architecture was developed for brain tumor classification. The architecture consists of four units. The first unit is the input layer, while the second unit consists of seven convolution layers stacked to each other. The seven-stacked convolutional layer is used to replace the single convolutional layer in the standard CapsNet. Each convolution layer is followed by the PSTanh activation function. The second unit is used to replace the standalone convolutional layer in standard CapsNet. The output feature map of the second unit is passed to the third unit. The third unit contains the primary capsule layer, while the last unit is the output capsule layer. The CapsNet model was evaluated on a brain tumor dataset (from Kaggle.com (accessed on 27 June 2022)) containing 3264 T_1_W images. Results show that the proposed PSTanh activation function outperforms the ReLU function by 7.11%, achieving a classification accuracy of 96.70%. The proposed activation function was compared to eight existing activation functions and was found to outperform them all.

#### 4.5.4. Brain Tumor Classification Using a Dilated Capsule Neural Network

The quality or resolution of an image is usually reduced during the feature extraction phase of CNN. The input images are typically downsampled to a point where the image loses its spatial recognizability. This loss can affect the classification performance of CapsNet. The loss can also affect scenarios where models are transferred to applications that require accurate and complete information of images. Adu et al. [109] developed a CapsNet-based technique using dilation with the objective of maintaining the high-resolution of the images for accurate classification. In the study, dilation was used to eliminate upsampling and to maintain high-resolution feature maps in the convolutional layers. More information on the dilated convolution is provided in [109]. The proposed framework consists of three convolutional layers, one primary capsule layer, one-digit capsule layer, and three fully-connected layers. The proposed technique was evaluated on a brain tumor dataset, and it produced a classification accuracy of 95.54%. The results also show that dilation reduced the network parameters and the training time.

#### 4.5.5. CapsNet-Based Image Pre-Processing Framework for Improved Brain Tumor Classification

Kurup et al. [110] presented a performance analysis on the effect of image pre-processing on CapsNet for brain tumor segmentation. They designed a CapsNet-based framework consisting of two main stages: image pre-processing and classification. The image pre-processing technique consists of two steps: rotation and patching. For image rotation, three different angles were used: 90°, 180°, and 270°. In addition, for patching, two patches of size 300 × 300 were extracted from each image. The augmented images and image patches were passed to CapsNet for classification. The CapsNet architecture comprises a convolutional layer, a primary capsule layer, a digit capsule layer, a fully connected layer, and an output layer with three neurons, one for each of the three output classes. The CapsNet was evaluated on the CE-MRI dataset. The dataset was used to train the CapsNet before and after image pre-processing. Results reported in the study show that the proposed technique produced an accuracy of 87% and 92.6%, before and after pre-processing, respectively. This shows that the pre-processing improved the accuracy of CapsNet by 5.6%.

#### 4.5.6. Brain Tumor Segmentation Using a Modified Version of Capsule Neural Network

Aziz et al. [37] introduced a CapsNet-based technique for segmenting brain tumors. They used the modified version of the original CapsNet architecture called SegCaps [42]. SegCaps was first proposed by Lalonde and Bagci [42] to address CapsNet’s [37] runtime and memory constraint issues. The routing method is one of the primary distinctions between CapsNet and SegCaps. In the SegCaps design, routing between lower layer capsules and upper layer capsules takes place entirely within a single spatial window, and transformation matrices are also shared between capsules of each layer. The proposed technique was evaluated on the BraTS2020 dataset. Twenty percent of the tumor slices were randomly selected and utilized to train SegCaps in the studies. Additionally, 85% of tumor slices were randomly chosen and used to train the U-Net architecture. Results show that SegCaps surpassed the U-Net architecture by 3%, obtaining a DSC of 87.96%. SegCaps accomplished this improvement by using 95.4% fewer parameters than U-Net.

**Figure 10:**
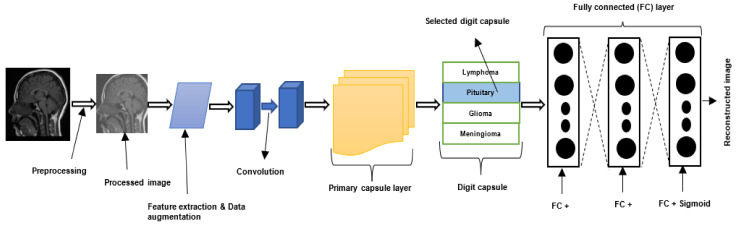
Workflow for a Capsule Network (CapsNet)-based brain tumor segmentation technique.

The workflow for a typical CapsNet-based brain tumor segmentation technique is shown in Figure 10. Moreover, the summary of all the CapsNet-based techniques reviewed in this study is presented in Table 6.

## 5. Discussion

Different ML-based, CNN-based, CapsNet-based, and ViT-based techniques have been developed so far. Generally, most of the techniques are divided into two stages: segmentation and classification stage. The segmentation stage is used to identify the specific region where the tumor is located and the subregions within the tumor, while the classification stage is used to identify the specific type or grade of the segmented tumor. Most of the techniques were developed to distinguish the different classes of CNS tumors including meningioma, pituitary tumors, glioma, and metastases. SVM, DT, NB, Bayesian algorithm, k-NN, ANNs, and CNN are the most widely used ML and DL algorithms for brain tumor classification and segmentation.

Brain tumors can be diagnosed using different types of medical images, such as CT and MRI. However, MRI is the reference standard in the clinical routine. This is due to the multiparametric nature of MRI. This section presents a discussion on the design and development of various brain tumor classification and segmentation techniques.

### 5.1. Machine Learning-Based Brain Tumor Classification and Segmentation Techniques

Brain tumor segmentation is basically a voxel-level classification task [10]. Therefore, classical ML algorithms are typically trained on voxel-based features extracted from different regions of interest [10,111]. ML algorithms are also trained on other types of features including tumor shape, image brightness, and Gabor features [112]. Some studies [15] introduced new features, such as texture-based features and Gabor features. Texture-based features can be used to identify different regions of interest in an image. Jena et al. [15] introduced seven texture-based features for classifying brain tumors. They combined the seven features to form a feature matrix and used the feature matrix to train five ML algorithms. Experiments performed in the study show that the texture-based features can effectively determine the location of tumors in an MRI scan.

Feature engineering is a very important step when building ML models. Manually designing robust features for ML algorithms can be very demanding and time-consuming. Therefore, some studies [13,14,84] used deep features to build brain tumor classification models. Deep features refer to features that are extracted from CNN models. These features are typically extracted from the convolutional and fully connected layers of pre-trained CNN models and used to train classical ML algorithms. Kang et al. [84] concatenated different deep features from pre-trained models and used the resultant feature maps to build ML models. Most of the pre-trained models were originally trained on a subset of the ImageNet dataset [113], consisting of over 1.2 million high-resolution labelled images in over 1000 classes. Some of the pre-trained network architectures that has been used in the literature include AlexNet [88], VGG [87], GoogleNet [83], ResNet [85], Inception-v3 [89], DenseNet [86], ShuffleNe-v2 [91], MobileNet-v2 [92], and MnasNet [93]. Results reported in the literature show that classical ML algorithms trained on deep features outperformed pre-trained models. For example, results reported by Sekhar et al. [14] show that GoogleNet produced precision and specificity of 96.02% and 96.00%, respectively using the softmax classifier. The results also show that the performance of GoogleNet was improved by over 2.5% and 2.3% when SVM and k-NN classifiers were used. This shows that the features extracted from CNN pre-trained models can be used to build effective ML-based models for brain tumor classification.

Training ML algorithms on many features will result in overfitting. Moreover, training ML algorithms on many features can be computationally expensive and may complicate the classification task. In some cases, classical ML algorithms yield better classification accuracy when trained on small-scale features compared to large-scale features. As a result, many studies used feature selection techniques to reduce the feature size used for training. PCA, LDA, and genetic algorithm (GA) are the most often used feature reduction techniques. Other feature selection techniques that are used in the literature include SVM-RFE and discrete wavelet transform (DWT). Sasikala and Kumaravel [114] introduced a wavelet-based feature selection technique, and results reported in the study indicated that the wavelet-based feature selection technique reduced the feature size by 83.34% (from 29 features to four features), and simultaneously achieved a classification accuracy of 98%.

### 5.2. Deep Learning-Based Brain Tumor Segmentation and Classification Techniques

Nowadays, DL approaches (such as CNNs) are acquiring greater prominence in the classification of brain images than ML techniques. In CNN, the images are used as direct input into the network. CNN and other DL methods generate translation-invariant and deformation-resistant features from images, resulting in more accurate segmentation. CNN is commonly used for brain image analysis in a variety of applications, including brain tumor segmentation and categorization. Most of the CNN network architecture designed in the literature can only effectively learn information from 2D MRI slices. They do not have the capacity to effectively extract volumetric information in 3D MRI slices. Mzoughi et al. [62] designed a 3D network architecture consisting of different 3D convolutional layers and small 3D kernels in each of the convolutional layers. The small 3D kernels (3 × 3) were used to improve the computational complexity of the network and reduce the number of weights in the network.

Some studies developed effective CNN-based techniques for brain tumor segmentation. AlBadawy et al. [73] introduced a patching-based technique for segmenting brain tumors obtained from multiple institutions. The results obtained in the study indicated that the performance of CNN declined when it was trained on brain scans from different institutions compared to the same institution. Havaei et al. [10] introduced two-pathway techniques that can both learn to segment small details (localized features) of brain tumors and global-scale features (contextual features). The first pathway uses 7 × 7 convolutional filters to learn the localized features and 13 × 13 to learn the global-scale features. The study also introduces a cascade architecture that can segment dependencies between adjacent labels. Finally, the study introduced a two-phase training strategy to handle the issue of data imbalance in many datasets. Results obtained in the study show that training the model on local and global features concurrently produced better outcomes than training separately and averaging the results.

A few authors [41,60] investigated the usage of small kernels with the goal of constructing deeper networks that do not overfit. Results achieved by the studies show that small kernels produce effective brain segmentation results. Furthermore, some studies designed effective multi-purpose frameworks for brain tumor diagnosis. For example, Sharif et al. [101] designed a multi-purpose framework that can perform different functions including tumor enhancement, feature extraction and selection, localization, and tumor segmentation. In the study, homomorphic wavelet filter, inception-v3 model, non-dominated sorted genetic algorithm (NGSA), YOLOv2, and ML algorithms were used for tumor enhancement, feature extraction, feature selection, localization, and tumor segmentation, respectively. Experiments performed in the study show that the multi-purpose framework produced good results. This type of framework will be very useful to medical practitioners, as it can help them to handle multiple tasks effectively.

Most of the CNN-based and CapsNet-based architectures developed in the literature used the ReLU activation function. The performance produced by ReLU-based networks can be improved [19]. Adu et al. [19] introduced a new activation function for improved tumor classification and segmentation, called PSTanh. Results reported in the study show that PSTanh activation function outperformed ReLU by 7.11%.

### 5.3. Vision Transformer-Based Tumor Segmentation and Classification

Most studies in the literature adopted the popular CNN-based “U-shaped” architecture. However, the kernel size of CNNs limits their ability to learn global or long-range information. Transformer architecture has been the de facto standard for natural language processing (NLP) [20]. However, its application to medical image processing is limited [20]. Some studies [32,35] developed ViT-based models for 2D or 3D image segmentation and classification. Some of them used ViT in conjunction with CNNs. They combined the advantage of CNNs and ViT to create models capable of capturing both local and global contextual representations. To be more precise, some studies [35,104] employed CNN architectures for both encoding and decoding. The CNN encoder is typically used to capture local contextual features. The captured features are passed into a ViT, which captures global semantic features. The global information is fed into a CNN decoder, which gradually upsamples them until a full resolution segmented image is recovered. Finally, a skip connection is employed to combine the features of the encoder and decoder network. Some studies [32,107] explored alternative approaches. Some of them used ViT for encoding and CNN for decoding [32], while others used ViT for both encoding and decoding [107]. The ViT-based encoders accept three-dimensional image patches as input. They directly process 3D volumetric data for semantic segmentation.

Transformers are quite difficult to apply to computer vision tasks. This is because of the distinctions between computer vision and NLP, such as the higher resolution of images compared to words and the wide range of scales of visual entities [106]. In light of this, Liu et al. [106] proposed Swin transformers to address this issue. Swin is a hierarchical transformer that computes its representation through the use of shifted windows [106]. The shifted window improves the performance of a model by restricting self-attention computation to non-overlapping windows while allowing cross-window communication [106]. Some studies [32] used Swin transformers to segment 3D multi-modal brain tumors and obtained promising results.

### 5.4. Capsule Neural Network-Based Brain Tumor Classification and Segmentation Techniques

Many CNN-based image classification techniques have been introduced in the literature, and most of them produced state-of-the-art results. CNNs can automatically learn high-level and low-level features from images without the need for feature engineering. However, CNNs require large datasets for training. Additionally, CNNs are incapable of correctly distinguishing between inputs of different rotations and transformations [40]. Hinton et al. [38] addressed these drawbacks by introducing CapsNet. CapsNet are very robust to different rotations and image transformation. Additionally, CapsNet require significantly less training data compared to CNN.

Different CapsNet-based brain tumor diagnosis techniques have been proposed in the literature, but most of them focused on brain tumor classification. Very few studies designed CapsNet-based techniques for brain tumor segmentation. CapsNet will be particularly useful for brain tumor segmentation because of their efficacy in object detection and image segmentation. They will also perform very well because they require small-scale datasets for training, which is the case for most of the benchmark datasets used for evaluating brain tumor classification and segmentation techniques. Moreover, CapsNet requires lesser computational complexity than CNNs. Aziz et al. [37] is one of the few studies that explored CapsNet for brain tumor segmentation. Results reported in the study [37] show that CapsNet outperformed CNN by 3%. In addition, results reported by Afshar et al. [18] show that CapsNet outperformed CNN by 14.43%. Moreover, experiments performed by Aziz et al. [37] demonstrate that CapsNet require significantly fewer parameters compared to CNNs. In their experiment, CapsNet used 95.4% fewer parameters than CNN and simultaneously outperformed CNN by 3% [37]. The experiments also show that CapsNet performs better when trained on the segmented tumor images compared to the whole brain MRI.

CNN algorithms normally use the max-pooling operation after a convolutional layer. The max-pooling operation is used to create downsampled feature maps that highlight the relevant feature maps in an image. However, downsampling can affect the quality or resolution of an image to a point where the image loses its spatial recognizability. This reduced image quality can affect the classification performance of CapsNet-based and CNN-based models. The loss can also affect scenarios where models are transferred to applications that require accurate and complete information of images. Adu et al. [109] tackled this problem by designing a CapsNet-based technique using dilation convolution to eliminate upsampling and to maintain high-resolution feature maps in the convolutional layers. Experiments performed in the study show that dilation reduced the number of network parameters and training time. The technique achieved a classification accuracy of 95.54%.

Traditional CNN and ML algorithms have the problem of model uncertainty. Afshar et al. [40] proposed a CapsNet-based technique that can handle model uncertainty. They used Bayesian theory to model the uncertainty associated with predictions of CapsNet models. Results reported in the study show that Bayesian theory improves the interpretability of a network, which is very important for medical applications.

### 5.5. Hybrid Brain Tumor Classification and Segmentation Techniques

Most studies [40,41,60,109] used one DL or ML algorithms to design tumor segmentation techniques. Few studies used a combination of different algorithms (i.e., hybrid techniques). Moreover, most studies used CNN or other popular ML algorithms, such as RF, SVM, and NB. Few studies explored the use of clustering algorithms for tumor segmentation. Jena et al. [15] designed a hybrid technique consisting of k-NN and fuzzy C-means clustering. They used the hybrid technique for tumor segmentation, and the experiment performed in the study shows that the hybrid technique produced promising results. Some researchers used the combination of bio-inspired techniques and CNN to design tumor segmentation techniques. Thaha et al. [17] used the BAT algorithm to optimize the loss function and consequently improve the segmentation accuracy of CNN. Results obtained showed that BAT algorithm improved the precision, recall (or sensitivity), and accuracy of CNN by at least 4%. Bio-inspired optimization algorithms are very good algorithms that can be combined with DL and ML algorithms to improve their performance.

Some researchers used ensemble-based classifiers to design tumor classification techniques. Ensemble-based classifiers are techniques that generate multiple models, which are then combined to produce improved results. Jena et al. [15] designed a tumor classification technique using a combination of seven algorithms, namely: Adaboost, Gentleboost, Logitboost, LPboost, Robustboost, RUSboost, and Totalboost. Results reported in the study show that the ensemble method produced the best classification accuracy compared to stand-alone classification algorithms, such as RF.

### 5.6. Small-Scale and Imbalance Dataset

CNN algorithms do not perform very well when they are trained on small-scale and imbalanced datasets. Unfortunately, most of the benchmark datasets used in the literature are small and very imbalanced. For example, 98% of the dataset used by Havaei et al. [10] belong to one class, while the remaining 2% belong to four other classes: necrosis, edema, non-ET, and ET. Obviously, training a CNN model on such imbalanced datasets will lead to overfitting. Some studies introduced techniques that can be used to solve the overfitting issue. Afshar et al. [18] used a regularization criterion (early stopping approach [31]) to handle the overfitting problems of CapsNet. Some studies [16,41,84] used data augmentation to handle small-scale and overfitting issues. Sajjad et al. [16] applied eight different data augmentation techniques to CNN for multi-grade brain tumor classification, including flipping, rotation, skewness, shear, sharpening, Gaussian blur, emboss, and edge detection. The techniques were applied to improve the geometric transformation and noise invariance of CNN. Results reported in the study [16] showed that data augmentation improved the classification accuracy of CNN from 90.03% to 95.5%.

### 5.7. Multi-Class and Binary Classification

One of the major challenges with existing classification techniques is binary classification [16]. Most of the existing techniques were developed to classify brain tumors into two classes, namely: benign and malignant. Sajjad et al. [16] is one of the few studies that developed a multi-grade classification technique. Robust multi-grade classification techniques can improve the decision-making and diagnosis of radiologists and other medical practitioners.

Yogananda et al. [74] noted that training a single network for multi-class segmentation is more time-consuming and computationally expensive compared to training three networks for individual binary segmentation. In view of this, Yogananda et al. [74] simplified the multiclass segmentation problem to a single binary segmentation problem and developed three distinct network architectures for each of the segmentation problems. The output of the three networks was concatenated using a triple volume fusion to create a segmentation volume with multiple classes. Experiments show that the proposed method produced promising results.

### 5.8. Network Architectures and Data Augmentation

Most of the studies used state-of-the-art network architectures to improve the accuracy and generalization performance of their results.

Most studies used the U-Net architecture. The U-Net architecture is a CNN architecture that was designed for fast and precise segmentation of biomedical images. The architecture is designed by Ronneberger et al. [115] to localize and distinguish borders by classifying each pixel, ensuring that the input and output have the same size. The U-Net architecture is comprised of nine blocks of layers. The first four blocks are referred to as the contracting or downsampling block/segment. It has two convolution layers and one max-pooling layer. The last four blocks are known as expansive or upsampling block. It has two convolution layers and one 2d convolutional layer that has been transposed. It is used to resize (or reconstruct) the feature map produced by the downsampling block to the original size of the input image, such that the size of the input image and output image are the same. This enables the architecture to do pixel-level image segmentation. The U-Net architecture was designed using Keras with Tensorflow as the backend [116,117].

In addition to the U-Net architecture, the following network architectures are widely used in the literature: ResNet, VGG-19, and InputCascadeCNN. The VGG-19 architecture is pre-trained on the ImageNet dataset [118] consisting of 1.2 million high-quality images. It consists of 16 convolutional layers and three fully connected layers. The InputCascadeCNN architecture [10] is designed to extract local and global contextual features. It consists of 7 × 7 feature maps for extracting local features (small details of the tumors) and 11 × 11 feature maps for extracting global features.

Some researchers introduced novel fine-tuning techniques for brain tumor classification and segmentation. Swati et al. [97] introduced a block-wise fine-tuning technique using the VGG-19 network architecture. They divided the layers of the VGG-19 network into different blocks and fine-tuned each block sequentially. While the layers in one block are fine-tuned, the layers in the other blocks are frozen. Experiments show that this technique produced promising results. Thus, more fine-tuning approaches can be developed to improve the performance of brain tumor diagnosis systems.

Most studies also applied data augmentation techniques to increase the dataset size and consequently improve the performance of CNN models. Some of the data augmentation techniques used in the literature include flipping, rotation, skewness, shears, sharpening, Gaussian blur, emboss, and edge detection. Some studies [16,95] used a combination of these techniques. Results reported in the literature show that data augmentation techniques improved the performance of DL-based brain tumor segmentation and classification models. In addition to data augmentation, some studies [75] used data enrichment techniques, such as GANs, to generate synthetic MR images. Many studies did not explore the use of advanced data enrichment techniques, and it is still an active research area.

### 5.9. Performance Overview of Brain Tumor Classification and Segmentation Techniques

**Figure 11:**
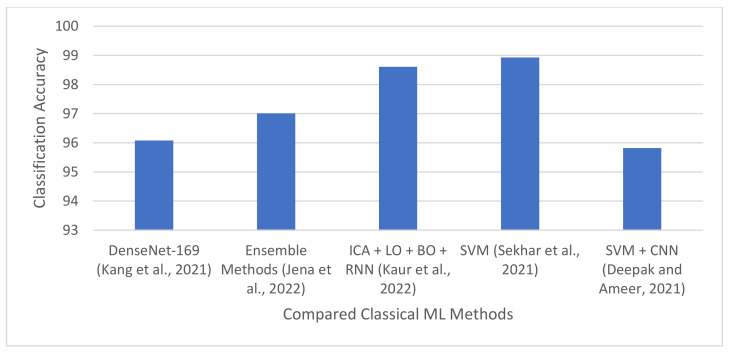
Summarized results for classical ML-based brain tumor classification techniques. For studies that used the same algorithm, we selected the algorithm that produced the best performance. The SVM model designed by Sekhar et al. [14] produced the best performance for ML-based tumor classification. The RNN constructed by Kaur et al. [94] generated the second-best result for ML-based brain tumor classification. The remaining competitors include the DenseNet-169 proposed in Kang et al. [84], the ensemble approach exploited by Jena et al. [15], and the work of Deepak and Ameer [13] that combined SVM with CNN.

**Figure 12:**
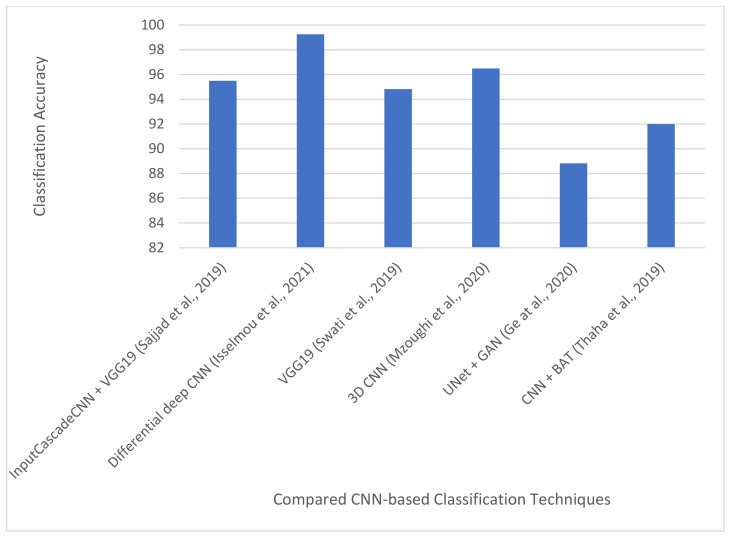
Performance overview of CNN-based brain tumor classification techniques. The technique developed by Isselmou et al. [95] achieved the best performance for CNN-based brain tumor classification. The techniques proposed by Mzoughi et al. [62] yielded the second-best classification accuracy. The remaining competitors include the work of Sajjad et al. [16], the VGG19-based approach of Swati et al. [97], the work of Ge et al. [75] based on UNet and GAN, and, finally, the combination of CNN and BAT proposed by Thaha et al. [17].

**Figure 13:**
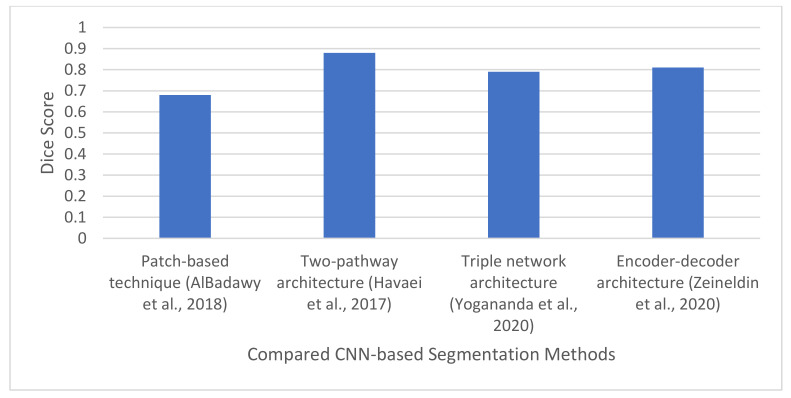
Performance overview of CNN-based brain tumor segmentation techniques. The technique developed by Havaei et al. [10] outperformed all the CNN-based brain tumor segmentation techniques presented in this study. The remaining competitors include the patching-based technique developed by AlBadawy et al. [73], the encoder-decoder architecture proposed by Zeineldin et al. [41], and the triple network architecture developed by Yogananda et al. [74].

**Figure 14:**
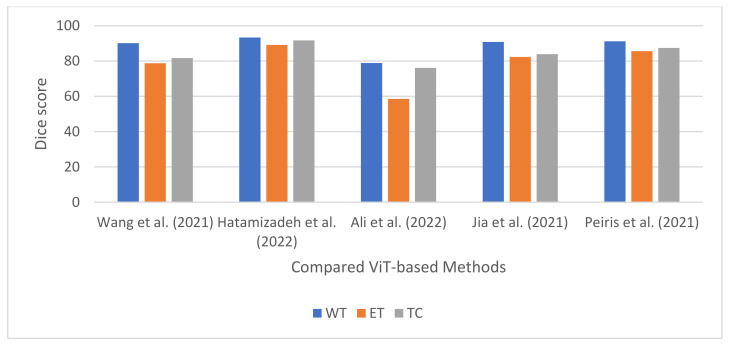
Performance overview of ViT-based techniques. The ViT-based technique developed by Hatamizadeh et al. [32] produced the best result, achieving a WT, ET, and TC of 93.3%, 89.1%, and 91.7%, respectively. The remaining competitors include the ViT-based technique developed by Jia et al. [104], the work of Peiris et al. [107], the study of Wang et al. [35], and the method proposed by Ali et al. [105].

**Figure 15:**
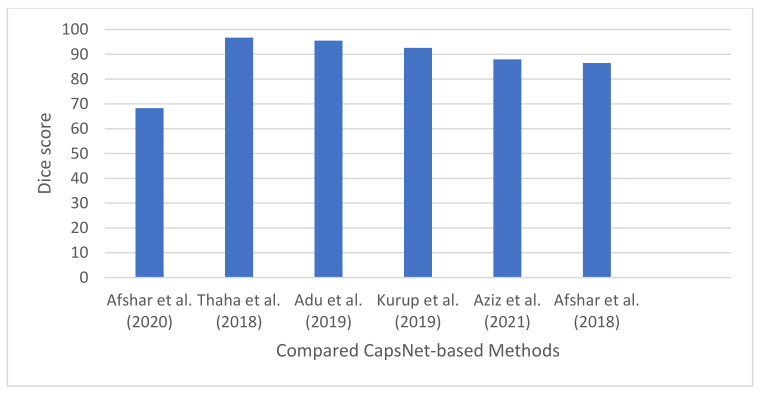
Performance overview of CapsNet-based brain tumor techniques. The technique proposed by Thaha et al. [17] in 2020 yielded the best result for CapsNet-based brain tumor segmentation and classification. The method developed by Adu et al. [19] produced the second-best result. The remaining competitors include the work of Afshar et al. [40], the studies of Kurup et al. [110] and Aziz et al. [37], and the method proposed by Afshar et al. [18] in 2018.

The performance of the brain tumor segmentation and classification techniques is highly dependent on several characteristics, including similarity measures, image content, and algorithm optimization. Figure 11, Figure 12, Figure 13, Figure 14 and Figure 15 show the performance of the segmentation and classification techniques reviewed in this study. For studies that used the same algorithm, we selected the algorithm that produced the best performance. As shown in Figure 11, the SVM model designed by Sekhar [14] produced the best performance for ML-based tumor classification. In the study [14], SVM was trained on features extracted from the GoogleNet pre-trained model [14]. The technique produced a classification accuracy of 98.93%. This shows that features extracted from CNN pre-trained models can be used to build good ML models with satisfactory accuracy. Figure 11 also shows that the RNN constructed by Kaur et al. [94] generated the second-best result for ML-based brain tumor classification. In this study, ICA was utilized for feature extraction, while a combination of the LO and BO algorithms was utilized for image smoothing. This highlights the need for feature extraction and image smoothing for traditional ML techniques.

Furthermore, as shown in Figure 12, the technique developed by Isselmou et al. [95] achieved the best performance for CNN-based brain tumor classification. Instead of using classical convolution feature maps, the authors [95] used user-defined hyperactive values and a differential operator to develop differential convolution maps. The technique achieved a classification accuracy of 99.25%. This demonstrates the potential of using differential feature maps for CNN models. Figure 12 also shows that the techniques proposed by Mzoughi et al. [62] yielded the second-best classification accuracy. Mzoughi et al. [62] developed a multi-scale CNN architecture with 3D convolutional layers for brain tumor grading. The authors also developed a technique for removing thermal noise and distortions in MRI images based on intensity normalization. This shows that 3D convolutional layers and intensity normalization can be used to design improved CNN-based brain tumor grade classification techniques.

Figure 13 shows the performance overview of the CNN-based brain tumor segmentation techniques reviewed in this paper. This plot shows that the technique developed by Havaei et al. [10] outperformed all the CNN-based brain tumor segmentation techniques presented in this study. The authors introduced a two-pathway architecture that can learn to segment both localized and global-scale features of the brain. This shows that building a model that can learn both local and global features concurrently will produce better outcomes compared to building separate models and averaging their results. Figure 13 also shows that the patching-based technique developed by AlBadawy et al. [73] produced the worst result, by achieving a DSC of 0.68.

Figure 14 shows the performance overview of the ViT-based techniques reviewed in this study. As shown in Figure 14, the ViT-based technique developed by Hatamizadeh et al. [32] produced the best result, achieving a WT, ET, and TC of 93.3%, 89.1%, and 91.7%, respectively. In the study [32], the authors developed a U-shaped architecture consisting of CNN and Swin. Moreover, the ViT-based technique developed by Jia et al. [104] produced the second-best result. The authors designed a UNet-based architecture for multi-modal MRI scans using CNN and ViT.

Figure 15 shows the results of the CapsNet-based brain tumor segmentation and classification techniques surveyed in this paper. As shown in this plot, the technique proposed by Thaha et al. [17] yielded the best result for CapsNet-based brain tumor segmentation and classification. The technique achieved a classification accuracy of 96.7%. In the study, the authors [17] developed a loss function optimization technique using the BAT algorithm. This demonstrates how bio-inspired algorithms can be used to improve CapsNet-based models. Figure 15 also shows that the method developed by Adu et al. [19] produced the second-best result. The authors [19] used a new activation function with CapsNet, and it achieved a classification accuracy of 95.54%.

## 6. Conclusions and Future Research Directions

The use of CNN methods to segment brain tumors is a worthwhile, but challenging, endeavor. Numerous ML and CNN approaches have been successfully applied in the literature to handle this difficult task. In this study, relevant ML-based, CNN-based, CapsNet-based, and ViT-based techniques for brain tumor classification and segmentation were examined, and a comprehensive survey of these techniques was presented accordingly. The possible future research directions are presented in what follows:

1. Most of the current research is devoted to brain tumor detection, segmentation, or grade estimation. Most studies did not develop frameworks that can perform these three tasks simultaneously. Moreover, most studies focused on binary-grade classification with less attention paid to multi-grade classification. Designing a framework that can handle brain tumor segmentation, tumor classification (benign versus malignant), and multi-grade estimation would be valuable in improving the decisions and accuracy of medical practitioners when diagnosing brain tumors.
2. Generally, feature maps in CNNs are generated using transfer learning or random initialization. Few studies developed techniques that can be used to generate feature maps for CNNs. Isselmou et al. [95] combined user-defined hyperactive values and a differential operator to generate feature maps for CNN. Results show that the differential feature maps improved the accuracy of the CNN model. Future research can develop more techniques that can generate effective feature maps for improved CNN-based brain tumor segmentation and classification.
3. Most of the existing DL brain tumor techniques are based on CNNs. However, these architectures require a huge quantity of data for training [9]. They are also incapable of correctly distinguishing between inputs of different rotations [10]. In addition, obtaining and labelling large-scale datasets is a demanding task [9]. Unfortunately, most publicly available brain cancer datasets are small and imbalanced. The accuracy and generalization performance of a CNN model will be affected if it is trained on small-scale or imbalanced datasets. CapsNet [11] is a recently developed network architecture that has been proposed to address the above-mentioned shortcomings of CNNs. CapsNet are particularly appealing because of their robustness to rotation and affine transformation. Additionally, as demonstrated in [2], CapsNets require significantly less training data than CNN, which is the case for medical imaging datasets such as brain MRI images [3]. Moreover, results reported in the literature [2] show that CapsNets have the potential to improve the accuracy of CNN-based brain tumor diagnosis using a very small number of network parameters [12]. Most studies did not explore the use of CapsNet for brain cancer diagnosis.
4. While ViT has demonstrated outstanding performance in NLP, its potential has not been fully explored for medical imaging analysis, such as brain tumor segmentation [33]. Future research can focus on improving the effectiveness of ViT-based approaches for classifying and segmenting brain tumors, as this is an active research area. Additionally, future research could further investigate the use of Swin transformers, as they seem to perform better than standard ViTs.
5. Most studies focused on 2D network architecture for tumor segmentation and classification. However, few studies explored 3D network architectures. 3D convolutional layers provide a detailed feature map that can explore the volumetric information in MRI scans [62]. The 3D feature map can also learn both local and global features with high classifying power [62]. Future researchers can explore 3D network architectures for improved brain tumor segmentation and classification.
6. Most of the techniques developed in the literature do not tackle the problem of model uncertainly in CNN-based and CapsNet-based models. Developing a network that can handle model uncertainty is important because it serves as a medium for returning the uncertain predictions to experts for further verification. Most CNN networks use the softmax activation function. However, the output of softmax does not reflect the degree of uncertainty of a model’s prediction [108]. Afshar et al. [40] developed a CapsNet-based technique that can handle model uncertainty using Bayesian theory. However, experiments performed in the study show that CapsNet (without Bayesian theory) outperformed the Bayesian variant. This confirms that more work is still required. Future research could focus on developing robust techniques that can effectively handle model uncertainty without affecting the performance of the models.
7. Most of the CNN-based and CapsNet-based architectures developed in the literature used the ReLU activation function. Few studies explored the use of other activation functions. Results reported by Adu et al. [19] show that PSTanh activation function outperformed the ReLU activation function. Future research could explore more activation functions for improved brain tumor classification and segmentation methods.
8. The max-pooling operation used in network architectures normally affects the image quality and resolution of an image, and consequently the accuracy of a network. Very few studies developed techniques that maintain high-resolution feature maps. Further work is therefore required in this area. Future research can develop effective techniques that can preserve the quality and spatial recognizability of an image.
9. Most of the currently available CNN-based approaches were developed for a specific form of cancer. A general-purpose DL-based framework that can diagnose different types of cancer will be very useful to medical practitioners
10. Most datasets cited in the literature suffer from data imbalance problems. For instance, 98% of samples in one of the benchmark datasets–Brain Tumor Segmentation (BraTS) dataset–belong to a single class, whereas the remaining 2% belong to another class. Clearly, building a model on such an imbalanced dataset will result in overfitting. Furthermore, most studies did not explore the use of advanced data enrichment methods, such as GANs, for improving the performance of brain tumor diagnosis. In addition, most studies did not investigate the performance of different data augmentation techniques for brain cancer diagnosis. Moreover, most studies did not investigate the use of different state-of-the-art pre-trained networks for brain cancer diagnosis. The problem of data imbalance and small-scale dataset in brain tumor diagnosis may be addressed by developing techniques that combine advanced data augmentation techniques and state-of-the-art pre-trained network architectures.
11. AlBadawy et al. [73] reported that there was a significant decrease in the performance of CNN models when they were trained for patients from the same institution compared to when they are trained for patients from different institutions. The reason behind the reduced performance requires systematic investigations.
12. The fusion of multi-modal data can improve the performance of brain tumor diagnosis models. Utilizing complementary information from multiple imaging modalities has sparked a rise in recent research interest in cross-modality MR image synthesis. Recent studies [119,120] have developed multi-modal brain tumor segmentation systems that can learn high-level features from multi-modal data. Future research can design enhanced multi-modal diagnostic frameworks for brain tumors.
13. Correct classification of multi-modal images of brain tumors is a vital step towards accurate diagnosis and successful treatment of brain tumors. However, resolving incomplete multi-modal issues is a challenging task in brain tumor diagnosis. Some techniques [121,122] have been proposed to address this difficulty, but more research is still required.
14. An important aspect that deserves further investigation is the development of integrative approaches, by considering clinical and multiomics data along with imaging [123,124], such as autoencoders [125] and variational autoencoders [126].

## Figures and Tables

**Table 1:** Summary of Dataset used in the literature.

Dataset Name	Dataset Details	Reference
BraTS 2012	30 MRI, 50 simulated images (25 Low Grade Glioma (LGG) and 25 High Grade Glioma (HGG))	[44]
BraTS 2013	30 MRI (20 HGG and 10 LGG), 50 simulated images (25 LGG and 25 HGG)	[45]
BraTS 2014	190 HGG and 26 LGG MRI	[46]
BraTS 2015	220 HGG and 54 LGG MRI	[47]
BraTS 2016	220 HGG and 54 LGG; Testing: 191 images with unknown grades	[48]
BraTS 2017	285 MRI scan. Contains full masks for brain tumors.	[49]
BraTS 2018	Training dataset: 210 HGG and 75 LGG MRI scans. The validation dataset includes 66 different MRI scans	[50]
BraTS 2019	259 HGG and 76 LGG MRI scans from 19 institution.	[51]
BraTS 2020	2640 MRI scans from 369 patients with ground truth in four sequences (T_1_-weighted (T_1_w), T_2_-weighted (T_2_w), post-gadolinium-based contrast agent (GBCA) T_1_w (T_1_w post GBCA), Fluid Attenuated Inversion Recovery (FLAIR))	[52]
BraTS 2021	8000 MRI scans from 2000 cases	[53]
TCIA	3929 MRI scans from 110 patients. 1373 tumor images and 2556 normal images.	[54]
Radiopedia	121 MRI	[55]
Contrast Enhanced Magnetic Resonance Images (CE-MRI) dataset	3064 MRI T_1_w post GBCA images from 233 patients	[56]
Brain MRI Images	253 MRI images, 155 tumor images, 98 non-tumor images	[57]
Br35H dataset	3000 MRI images, 1500 tumor images, and 1500 non-tumor images	[58]
MSD dataset	484 multi-modal multi-site MRI data (FLAIR, T_1_W, T_1_w post GBCA, T_2_W)	[59]

**Table 2:** Summary of classical ML-based techniques.

Ref.	Year	Method	Classes Considered	Main Highlight	Dataset	Performance
[84]	2021	13 pre-trained CNN models and nine ML classifiers	Normal and tumor images	Concatenated three deep features from pre-trained CNN models and trained nine ML classifiers.	Three brain MRI datasets [56,57,58]	DenseNet-169, Inception-v3, and ResNeXt-50 produced the best deep features, achieving an accuracy of 96.08%, 92.16% and 94.12%, respectively.
[14]	2021	SVM, k-NN, and modified GoogleNet pre-trained architecture	Glioma, meningioma, and pituitary.	Extracted features from modified GoogleNet pre-trained architecture and used it to train SVM and k-NN	CE-MRI dataset	SVM and k-NN produced a specificity of 98.93% and 98.63%, respectively.
[15]	2022	SVM, k-NN, binary decision tree, RF, ensemble methods.	FLAIR, T_1_w, T_1_w post GBCA, and T_2_w	Developed multiple ML models on six texture-based features. Applied a hybrid of k-NN and C-means clustering for tumor segmentation.	BraTS 2017 and BraTS 2019	Classification accuracy of 96.98% and 97.01% for BraTS 2017 and 2019, respectively. DSC and accuracy of 90.16% and 98.4%, respectively.
[94]	2022	NB, RNN, bat and lion optimization, PCA, ICA, and cuckoo search.	Tumor and normal images	Designed hybrid techniques model using the combination of metaheuristics and ML algorithms.	TCIA	Classification accuracy of 98.61%.
[13]	2021	SVM and CNN	Glioma, meningioma, and pituitary tumor	Proposed a hybrid technique using CNN-based features and SVM.	FigShare dataset	95.82% accuracy

**Table 3:** Summary of CNN-based classification techniques.

Ref.	Year	Classes Considered	Method	Main Highlight	Dataset	Performance
[16]	2019	Grades I–IV	InputCascadeCNN + data augmentation + VGG-19	Adopted four data augmentation techniques. Also used InputCascadeNN architecture for data augmentation and VGG-19 for fine-tuning.	Radiopedia and Brain tumor dataset	Classification accuracy of 95.5%, 92.66%, 87.77%, and 86.71%. for Grades I–IV, respectively on radiopedia dataset. Sensitivity and specificity 88.41% and 96.12%, respectively, on the brain tumor dataset.
[95]	2021	T_1_w, T_2_w and FLAIR images	Differential deep CNN + Data augmentation	Applied user-defined hyperactive values and a differential operator to generate feature maps for CNN. Proposed several data augmentation techniques.	TUCMD (17,600 MR brain images)	Classification accuracy, sensitivity, and specificity of 99.25%, 95.89%, and 93.75%, respectively
[97]	2019	Glioma, meningioma, and pituitary tumor	VGG-19	Introduced a block-wise fine-tuning technique for multi-class brain tumor MRI image.	CE-MRI [56] 3064 images from 233 patients.	Classification accuracy: 94.82%
[62]	2020	LGG and HGG	3D CNN	Proposed a multi-scale 3D CNN architecture for grade classification capable of learning both local and global brain tumor features. Applied two image pre-processing techniques for reducing thermal noise and scanner-related artifacts in brain MRI. Used data augmentation.	BraTS2018: Training-209 HGG and 75 LGG from 284 patients. Validation: 67 mixed grades.	Classification accuracy: 96.49%
[75]	2020	T_1_w, T_1_w post GBCA, T_2_w, FLAIR	U-Net architecture, GANs	GANs was used to generate synthetic images for four modalities of MRI: T_1_, T1e, T_2_, FLAIR.	TCGA-GBM [99] and TCGA-LGG [99].	Average classification accuracy, sensitivity, and specificity of 88.82%, 81.81%, and 92.17%, respectively
[17]	2019	Complete, core, and enhancing tumors	Custom CNN architecture, Bat algorithm	Used BAT algorithm to optimize the loss function of CNN. In addition, used skull stripping and image enhancement techniques for image pre-processing.	BraTS2015	Accuracy, recall (or sensitivity), and precision of 92%, 87%, and 90%, respectively

**Table 4:** Summary of CNN-based segmentation techniques.

Ref.	Year	Classes Considered	Method	Main Highlight	Dataset	Performance
[73]	2018	Metastasis, meningiomas gliomas	CNN	Designed a patching-based technique for brain tumor segmentation. Evaluated the impact of inter-institutional dataset.	TCIA	DSC-Same institution: 0.72 ± 0.17 and 0.76 ± 0.12. Different Institution: 0.68 ± 0.19 and 0.59 ± 0.19
[10]	2017	Necrosis, edema, non-ET, ET.	CNN	Designed a two-pathway architecture for capturing global and local features. Also designed three cascade architectures.	BraTS2013	DSC: 0.88
[74]	2020	WT, TC, and ET	Triple CNN architecture for multi-class segmentation.	Developed a triple network architecture to simplify the multiclass segmentation problem to a single binary segmentation problem.	BraT2018 dataset	DSC: 0.90, 0.82, and 0.79 for WT, TC, and ET, respectively
[41]	2020	T_1_w, T_1_w post GBCA, T_2_w, and FLAIR	Modified U-Net architecture, Data augmentation, batch normalization using the N3 bias correction tool [100].	Developed an encoder-decoder architecture for brain tumor segmentation.	BraTS 2019	DSC, sensitivity, and specificity of 0.814, 0.783, 0.999, respectively.
[101]	2021	HGG and LGG	Inception-v3, NSGA, LDA, SVM, k-NN, softmax, CART, YOLOv2, and McCulloch’s Kapur entropy	Designed a CNN-based hybrid framework for tumor enhancement, feature extraction and selection, localization, and tumor segmentation	BraTS 2018, BraTS2019, and BraTS2020	Classification accuracy of 98%, 99%, and 99% for BraTS2018, BraTS2019, and BraTS2020, respectively.
[17]	2019	Complete, core, and enhancing tumors	Custom CNN architecture, Bat algorithm	Used BAT algorithm to optimize the loss function of CNN. In addition, used skull stripping and image enhancement techniques for image pre-processing.	BraTS2015	Accuracy, recall (or sensitivity), and precision of 92%, 87%, and 90%, respectively

**Table 5:** Summary of ViT-based techniques.

Ref.	Year	Classes Considered	Method	Dataset	Main Highlight	Performance
[35]	2021	ET, WT, EC	Transformers and 3D CNN	BraTS2019 and BraTS2020	Developed a transformer-based network for 3D brain tumor segmentation.	BraTS2020-DSC of 90.09%, 78.73%, and 81.73% for WT, ET, and TC, respectively. BraTS2019–DSC of 90%, 78.93%, and 81.84% for WT, ET, and TC, respectively DSC of 90%, 78.93%, and 81.84% for WT, ET, and TC, respectively
[32]	2022	ET, WT, and TC	Swin transformers and CNN	BraTS2021	Developed a technique for multi-modal brain tumor images using Swin transformers and CNN.	DSC of 0.891, 0.933, and 0.917 for ET, WT, and TC, respectively.
[105]	2022	WT, ET, and TC	Transformers and CNN	MSD dataset	Developed a segmentation technique for multi-modal brain tumor image using transformers and CNN.	DSC of 0.789, 0.585,, and 0.761 for WT, ET, and TC, respectively.
[104]	2021	WT, ET, and TC	Transformers and 3D CNN	BraTS2021	Designed a CNN-transformer technique for multi-modal brain MRI scan segmentation.	DSC of 0.823, 0.908, and 0.839 for ET, WT, and TC, respectively.
[107]	2021	WT, ET, and TC	Transformers and 3D CNN	BraTS2021	Developed a U-Net shaped encoder-decoder technique using only transformers. The transformer encoder can capture local and global information. The decoder block allows parallel computation of cross- and self-attention.	DSC of 85.59%, 87.41%, and 91.20% for ET, TC, and WT, respectively

**Table 6:** Summary of CapsNet-based brain tumor segmentation module.

Ref.	Year	Classes Considered	Method	Dataset	Main Highlight	Performance
[40]	2020	Meningioma, glioma, and pituitary.	CapsNet and Bayesian theory.	Cancer dataset [56]	Designed a DL technique that can model uncertainty associated with predictions of CapsNet models.	Classification accuracy: 68.3%
[19]	2021	Meningioma, Glioma, Pituitary, normal	CapsNet	Brain tumor dataset. Meningioma (937 images), Glioma (926 images), Pituitary (901 images), normal (500)	Introduced a new activation function for CapsNet, called PSTanh activation function.	Classification accuracy of 96.70%.
[109]	2019	Meningioma, Glioma, Pituitary, normal	CapsNet, dilation convolution	Brain tumor dataset [56]. 3064 images from 233 patients. Meningioma (708 slices), Glioma (1426 slices), Pituitary (930 slices), normal	Developed a CapsNet-based technique using dilation convolution with the objective of maintaining the high resolution of the images for accurate classification.	Classification accuracy: 95.54%.
[110]	2019	Meningioma, Glioma, Pituitary	CapsNet; classification; Data pre-processing	Brain tumor dataset: 3064 [56].	Presented a performance analysis on the effect of image pre-processing on CapsNet for brain tumor segmentation.	Classification accuracy: 92.6%
[37]	2021	T_1_w, T_2_w, T_1_ w post GBCA and FLAIR	SegCaps–Capsule network; brain tumor segmentation	BraTS 2020	Designed a modified version of CapsNet using SegCaps network.	DSC of 87.96%.
[18]	2018	Meningioma, Pituitary, and Glioma	Capsule network	Brain tumor dataset proposed by [56]	Developed different CapsNet for brain tumor segmentation. Investigated the performance of input data on capsule network. Developed a visualization paradigm for the output of capsule network.	86.5% for segmented tumor, and 78% for whole brain image

## Data Availability

Not applicable.
